# Retrograde Transport by Clathrin-Coated Vesicles is Involved in Intracellular Transport of PrP^Sc^ in Persistently Prion-Infected Cells

**DOI:** 10.1038/s41598-018-30775-1

**Published:** 2018-08-16

**Authors:** Takeshi Yamasaki, Akio Suzuki, Rie Hasebe, Motohiro Horiuchi

**Affiliations:** 1 Laboratory of Veterinary Hygiene, Faculty of Veterinary Medicine, Graduate School of Infectious Diseases, Hokkaido University, Kita 18, Nishi 9, Kita-ku, Sapporo, 060-0818 Japan; 2 Global Station for Zoonosis Control, Global Institute for Collaborative Research and Education, Hokkaido University, Kita 20, Nishi 10, Kita-ku, Sapporo, 001-0020 Japan

## Abstract

Intracellular dynamics of an abnormal isoform of prion protein (PrP^Sc^) are tightly associated with prion propagation. However, the machineries involved in the intracellular trafficking of PrP^Sc^ are not fully understood. Our previous study suggested that PrP^Sc^ in persistently prion-infected cells dynamically circulates between endocytic-recycling compartments (ERCs) and peripheral regions of the cells. To investigate these machineries, we focused on retrograde transport from endosomes to the trans-Golgi network, which is one of the pathways involved in recycling of molecules. PrP^Sc^ was co-localized with components of clathrin-coated vesicles (CCVs) as well as those of the retromer complex, which are known as machineries for retrograde transport. Fractionation of intracellular compartments by density gradient centrifugation showed the presence of PrP^Sc^ and the components of CCVs in the same fractions. Furthermore, PrP^Sc^ was detected in CCVs isolated from intracellular compartments of prion-infected cells. Knockdown of clathrin interactor 1, which is one of the clathrin adaptor proteins involved in retrograde transport, did not change the amount of PrP^Sc^, but it altered the distribution of PrP^Sc^ from ERCs to peripheral regions, including late endosomes/lysosomes. These data demonstrated that some PrP^Sc^ is transported from endosomes to ERCs by CCVs, which might be involved in the recycling of PrP^Sc^.

## Introduction

Prions are the causative agents of transmissible spongiform encephalopathies (TSEs), which are neurodegenerative disorders that are characterized by the accumulation of an abnormal isoform of prion protein (PrP^Sc^) in the central nervous system (CNS). PrP^Sc^ is the only known proteinaceous component of prions, and the infectivity of prions is thought to be associated with PrP^Sc^ oligomers^1,2^. PrP^Sc^ is generated from a cellular isoform of prion protein (PrP^C^) that is encoded by the *Prnp* gene of the host^3^. The generation of PrP^Sc^ in neurons is considered to be closely associated with neurodegeneration in prion diseases^4–6^; therefore, the cellular mechanism of PrP^Sc^ formation should be elucidated in order to understand the mechanism of neurodegeneration within prion diseases.

The intracellular dynamics of PrP^Sc^ in cells persistently infected with prions have been analyzed in order to investigate the mechanisms of PrP^Sc^ formation. Previous studies have shown that PrP^Sc^ localizes throughout the intracellular compartments, specifically the plasma membrane, early endosomes, recycling endosomes, late endosomes, lysosomes, and the perinuclear Golgi region^7–13^. Earlier studies suggested that the generation of PrP^Sc^ occurs on the cell surface or within the endocytic pathway^14–16^. Recent studies reported that the endocytic-recycling compartments (ERCs)^12^ and/or multivesicular bodies (MVBs)^17^ may be the sites where the conversion of PrP^C^ to PrP^Sc^ occurs. Our recent data also suggested that both the endocytic-recycling and endolysosomal pathways are involved in PrP^Sc^ formation^18^. In addition, a recent report suggested that certain intracellular trafficking, especially retrograde transport via retromers, is involved in the degradation of PrP^Sc^ within cells^19^. Taken together, the intracellular dynamics of PrP^Sc^ along with membrane trafficking are closely associated not only with the generation of PrP^Sc^ but also with the degradation of PrP^Sc^.

Considering that PrP^Sc^ is generated from PrP^C^ at the endocytic compartments along with membrane trafficking, it is important to clarify which machineries are responsible for the trafficking of PrP^Sc^. It is reported that newly synthesized PrP^Sc^ at the cell surface is rapidly internalized into early endosomes and transported to the endocytic-recycling pathway or endolysosomal pathway^19^. Considering that PrP^Sc^ is found in clathrin-coated pits at the plasma membrane^11^, PrP^Sc^ on cell surfaces may be internalized via clathrin-dependent endocytosis. Recent studies showed that some part of the internalized PrP^Sc^ is sorted by the retromer complex within early or late endosomes^17,19^. However, it is not clear whether the destination of PrP^Sc^ transported by the retromer complex is to either the retrograde pathway for recycling or the endolysosomal pathway for degradation. Our previous study suggests that PrP^Sc^ dynamically circulates between ERCs and peripheral regions, including the plasma membrane, via the endocytic-recycling pathway^13^. We also showed that the redistribution of PrP^Sc^ from the endocytic-recycling pathway to the endolysosomal pathway resulted in the degradation of PrP^Sc^ in lysosomes^20^. Although sorting PrP^Sc^ away from the degradative pathway and toward the recycling pathway is considered to be important for continuous generation of PrP^Sc^, the machinery involved in the recycling of PrP^Sc^ remains unknown.

Retrograde transport from endosomes to the TGN is one of the pathways involved in the recycling of molecules, such as cation-independent mannose 6-phosphate receptor (CI-MPR)^21^, trans-Golgi network protein (Tgn38)^22^, and TGN-resident protease furin^23^, which are known to circulate between the TGN and the plasma membrane through endosomes^24^. The retrograde transport from endosomes to the TGN is also used for trafficking of bacterial toxins, such as Shiga and cholera toxins, in order to mediate cytotoxicity. Shiga toxin B subunit (STxB) and cholera toxin B subunit (CTxB) bind globotriaosylceramide and GM1 ganglioside at the cell surface^25^, respectively, and are internalized into early endosomes and transported to the TGN via retrograde transport^26,27^. In our previous study, we showed that PrP^Sc^ in persistently prion-infected cells shared the endocytic pathway with exogenously introduced STxB and CTxB that passed through ERCs during their retrograde transport from early endosomes to the TGN^13^. These facts raised the hypothesis that PrP^Sc^ is transported to ERCs by a certain cellular machinery for the retrograde transport from endosomes to the TGN in a similar manner to cargo proteins such as STxB and CTxB. In the present study, we analyzed the localization of PrP^Sc^ and membrane trafficking machineries associated with the retrograde transport from endosomes to the TGN. We found that PrP^Sc^ is present within clathrin-coated vesicles (CCVs) in the cells, and at least part of the PrP^Sc^ is transported from peripheral endosomal compartments to ERCs by CCVs.

## Results

### Intracellular Localization of PrP^Sc^ and Components of Machineries for Retrograde Transport

**Figure 1:**
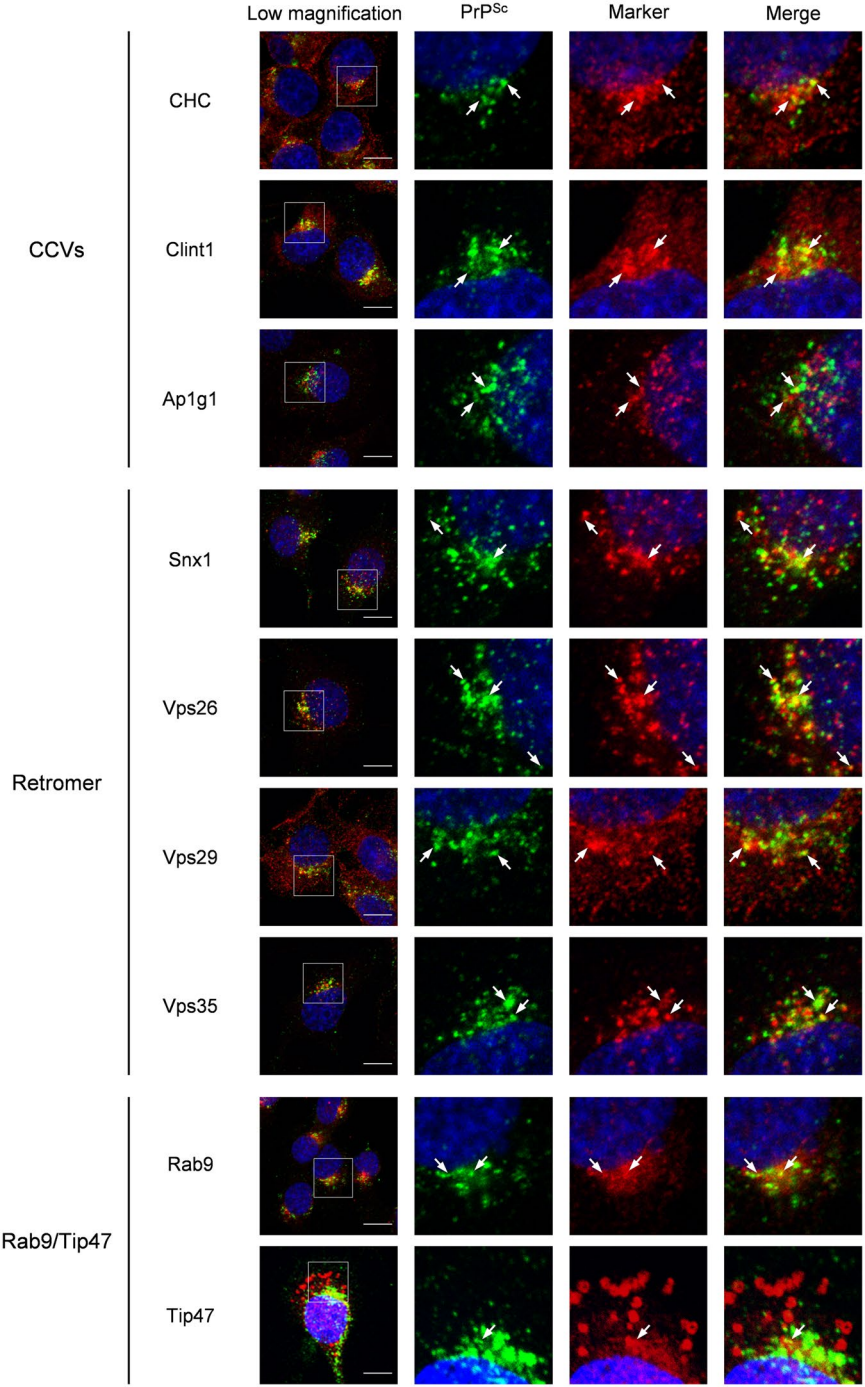
Co-localization of PrP^Sc^ with the components of machineries involved in retrograde transport from the endosomes to the TGN in ScN2a-3-22L cells. ScN2a-3-22L cells grown on the Chambered Coverglass for 72 h were subjected to IFA. The cells were stained with mAb 132 for PrP^Sc^, antibodies against CHC, Clint1, and Ap1g1 (component molecules of CCVs), antibodies against Snx1, Vps26, Vps29, and Vps35 (component molecules of the retromer complex), and antibodies against Rab9 and Tip47 (component molecules of the Rab9/Tip47 pathway). The cell nuclei were counterstained with DAPI. The leftmost column shows merged images of PrP^Sc^ (green), with the component molecules indicated on the left (red), and nuclei (blue). The other three images in the row correspond to high-magnification images of the boxed region for the merged image of PrP^Sc^ and nuclei (second left), the merged image of the component molecules and nuclei (second right), and the merged images of PrP^Sc^ with the component molecules and nuclei (far right). The arrows indicate representative examples of areas where PrP^Sc^ and the corresponding component molecules are co-localized. The co-localization areas were defined as pixels that were positive for both PrP^Sc^ signals and signals of the corresponding component molecules. The ratios of PrP^Sc^ signals co-localized with the signals of corresponding component molecules relative to the total signals of PrP^Sc^ are shown in Supplementary Fig. S1a. Scale bars: 10 µm.

Retrograde transport can occur within different stages of endosomes (early, recycling, or late) with the aid of membrane coat proteins for the proper transport of specific cargo proteins^26^. The best characterized coat proteins involved in the transport from endosomes to the TGN are the clathrin and retromer complex. Gamma1-adaptin (Ap1g1), a subunit of adaptor protein 1 (Ap1), and clathrin interactor 1 (Clint1) are known as clathrin adaptor proteins that mediate the transport of STxB and CTxB from early or recycling endosomes to TGN via CCVs^28,29^. Sorting nexin 1 (Snx1) and vacuolar protein sorting (Vps) 26, Vps29, and Vps35 are known as components of the retromer complex that also mediate the retrograde transport of STxB^30,31^. In addition, Tip47, which is one of the effectors of Rab9, is considered to act as a coat-like protein that mediates trafficking of CI-MPR from late endosomes to the TGN^26^. In order to clarify the association of these machineries with PrP^Sc^ transport, we analyzed the co-localization of PrP^Sc^ with the components of CCVs, those of the retromer complex, and Rab9/Tip47 in persistently prion-infected cells by the immunofluorescence assay (IFA) (Fig. 1). In a subclone of Neuro2a cells persistently infected with 22 L prion (ScN2a-3-22L cells), PrP^Sc^ at the perinuclear region was partially co-localized with the components of CCVs—clathrin heavy chain (CHC), Ap1g1, and Clint1—as well as the components of the retromer complex: Snx1, Vps26, Vps29, and Vps35. PrP^Sc^ was also co-localized with Rab9 at the perinuclear region but barely co-localized with Tip47. In order to confirm the co-localization of PrP^Sc^ with the components of CCVs, those of the retromer complex, and Rab9, we performed quantitative co-localization analysis using three-dimensional (3D) images of ScN2a-3-22L cells (Supplementary Fig. S1a). Ratios of PrP^Sc^ signals co-localized with the signals of CHC, Clint1, Ap1g1, Snx1, Vps26, Vps29, Vps35 and Rab9 relative to the total PrP^Sc^ signals were 13%, 8%, 29%, 14%, 9%, 14%, 11%, and 13%, respectively, which confirmed the partial co-localization of PrP^Sc^ with these component molecules.

**Figure 2:**
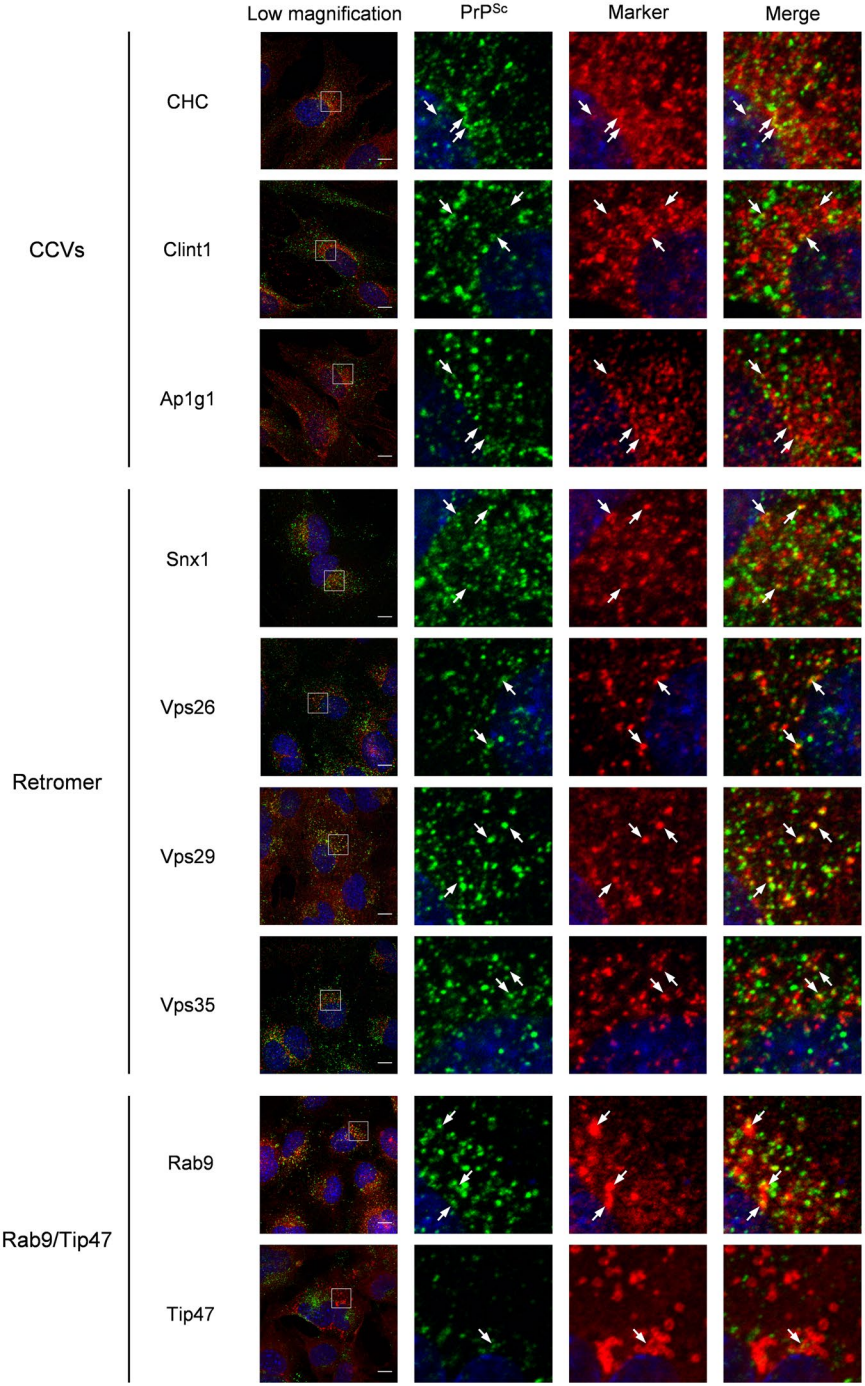
Co-localization of PrP^Sc^ with the components of machineries involved in retrograde transport from the endosomes to the TGN in ScGT1-7-22L cells. ScGT1-7-22L cells grown on the Chambered Coverglass for 96 h were subjected to IFA to monitor PrP^Sc^ and the component molecules of CCVs, as well as those of the retromer complex and Rab9/Tip47 pathway. The leftmost column shows lower magnifications of merged images of PrP^Sc^ (green), the component molecules (red), and nuclei (blue). Individual and merged high-magnification images of the boxed regions are shown on the right. The arrows indicate representative examples of areas where PrP^Sc^ and the corresponding component molecules are co-localized. The co-localization areas were defined as pixels that were positive for both PrP^Sc^ signals and signals of the corresponding component molecules. The ratios of PrP^Sc^ signals co-localized with the signals of corresponding component molecules relative to the total signals of PrP^Sc^ are shown in Supplementary Fig. S1b. Scale bars: 10 µm.

In order to confirm the association of PrP^Sc^ with the machineries for the retrograde transport in another cell line, we analyzed the co-localization of PrP^Sc^ with the components of CCVs, those of the retromer complex and Rab9/Tip47 (Fig. 2), as well as the co-localization of PrP^Sc^ with several markers of organelles (Supplementary Fig. S2) in GT1-7 cells persistently infected with 22 L prion (ScGT1-7-22L cells), as we previously reported in ScN2a-3-22L cells^13^. In ScGT1-7-22L cells, PrP^Sc^ was partially co-localized with Rab4a, Rab5a, Rab11a, and exogenously introduced transferrin (Tfn), which are the markers of organelles in the endocytic-recycling pathway, at the perinuclear region (Supplementary Fig. S2). PrP^Sc^ was partially co-localized with Rab7, Lamp1, and cathepsin D, which are the markers of organelles in the endolysosomal pathway, at relatively peripheral regions of the cells. In contrast, only a small portion of PrP^Sc^ was co-localized with Tgn38, a marker of the TGN, and giantin, a marker of the cis/medial Golgi, which are organelles in the secretory pathway. This result suggests that PrP^Sc^ is mainly localized at organelles in the endocytic-recycling and endolysosomal pathways, but not at organelles in the secretory pathway, which is consistent with our previous study using ScN2a-3-22L cells^13^. PrP^Sc^ in ScGT1-7-22L cells was partially co-localized with the components of CCVs and the retromer complex and Rab9 but barely co-localized with Tip47 in a similar manner to ScN2a-3-22L cells (Fig. 2). The quantitative co-localization analysis using 3D images confirmed the partial co-localization of PrP^Sc^ with the components in ScGT1-7-22L cells (Supplementary Fig. S1b).

### Subcellular Localization of PrP, Components of CCVs, and the Retromer Complex

**Figure 3:**
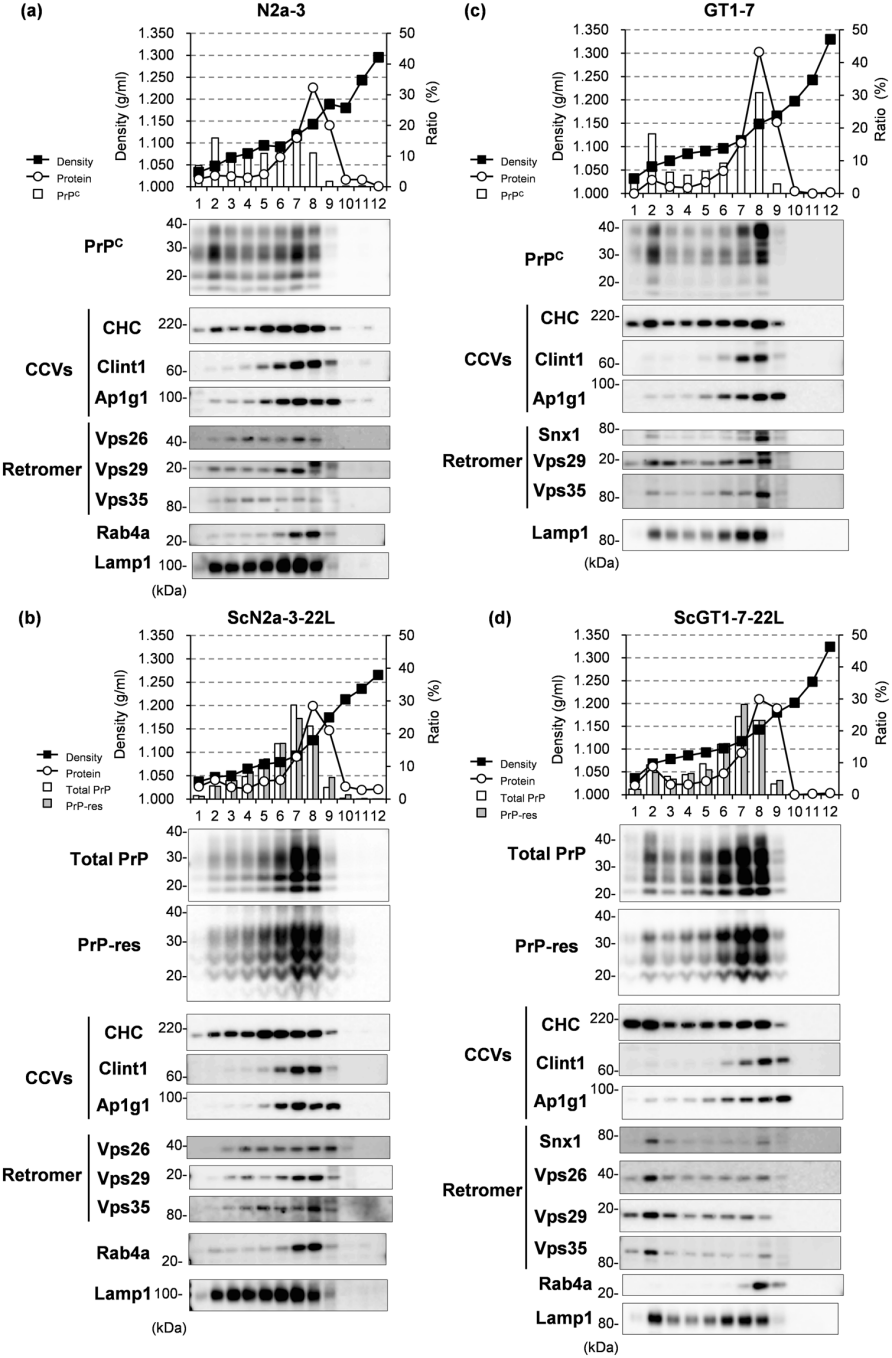
Subcellular localization of PrP^C^ and PrP^Sc^ and the component molecules of CCVs and the retromer complex. PNS fractions were prepared from N2a-3 cells (**a**), ScN2a-3-22L cells (**b**), GT1-7 cells (**c**), and ScGT1-7-22L cells (**d**) and were then separated into 12 fractions from the top to the bottom (numbered from 1 to 12) after density gradient centrifugation with iodixanol solution. Each fraction was subjected to measurement of density, analysis of protein concentration, and immunoblot analysis to monitor PrP and PrP-res, the component molecules of CCVs and the retromer complex, Rab4a, and Lamp1. The graphs on the top show the densities of the fractions (black squares), the ratios of the protein amount in each fraction to the total amount of protein in the 12 fractions (open circles), PrP^C^ or total PrP levels relative to the total amount of PrP in the 12 fractions (white bars), and PrP-res levels relative to the total amount of PrP-res in the 12 fractions (gray bars). The figures below show images of immunoblot assays for PrP, the components of CCVs; CHC, Clint1, and Ap1g1, those of the retromer complex; Snx1, Vps26, Vps29, and Vps35, and Rab4a and Lamp1. The cropped blots are shown in the figure, and full-length blots are presented in Supplementary Figs S6, S7, S8, and S9.

In order to further explore the association of PrP^Sc^ with the components of CCVs and the retromer complex, we analyzed their subcellular localizations (Fig. 3). Postnuclear supernatant (PNS) was prepared from N2a-3, ScN2a-3-22L, GT1-7, and ScGT1-7-22L cells and pelleted by ultracentrifugation to remove the cytosolic fraction. The PNS was subsequently separated into 12 fractions by iodixanol density gradient centrifugation. After the densities of iodixanol and the protein amount of each fraction were measured to confirm successful separation, the fractions were analyzed for PrP, the components of CCVs and the retromer complex, Rab4a, and Lamp1 by immunoblotting. An early endosome marker Rab4a was mainly distributed to high density fractions (Fig. 3a,b and d, Fractions 7–9), while a late endosome/lysosome marker Lamp1 was distributed through fractions 2–8 (Fig. 3a–d), suggesting that the intracellular compartments including the endosomes could be separated by their densities. PrP^C^ in N2a-3 (Fig. 3a) and GT1-7 cells (Fig. 3c) was distributed through fractions 1–9 and was mainly present at a low density fraction (Fraction 2) and at high density fractions (Fractions 6–8), separately. Total PrP and proteinase K- (PK-) resistant PrP^Sc^ (PrP-res) in ScGT1-7-22L cells were partially distributed to the low density fraction but were more distributed to the high density fractions (Fig. 3d). In contrast, total PrP and PrP-res in ScN2a-3-22L cells were mainly present not in the low density fraction, but within the high density fractions (Fig. 3b). In ScN2a-3-22L cells, the components of the retromer complex—Vps26, Vps29, and Vps35—were broadly distributed through low to high density fractions (Fig. 3b, Fractions 2–8). In ScGT1-7-22L cells, Snx1, Vps26, Vps29, and Vps35 were mainly distributed in the low density fraction (Fig. 3d, Fraction 2), where only a small amounts of total PrP and PrP-res were present. Both in ScN2a-3-22L and ScGT1-7-22L cells, CHC was distributed to both the low and the high density fractions (Fig. 3b,d). However, Clint1 and Ap1g1 were mainly present within the high density fractions (Fig. 3b,d, Fractions 6–8 of ScN2a-3-22L cells and Fractions 7–9 of ScGT1-7-22L cells), where higher amounts of total PrP and PrP-res were present compared to low density fractions (Fractions 1–3 of ScN2a-3-22L and ScGT1-7-22L cells). The difference in the subcellular distribution between PrP-res and the components of the retromer complex, but the subcellular co-localization of PrP-res with the components of CCVs at high density fractions in ScN2a-3-22L and ScGT1-7-22L cells suggests that PrP^Sc^ is mainly associated with CCVs rather than the retromer complex, and that the association of PrP^Sc^ with CCVs is common event over the cell type.

### Presence of PrP^Sc^ in CCVs

**Figure 4:**
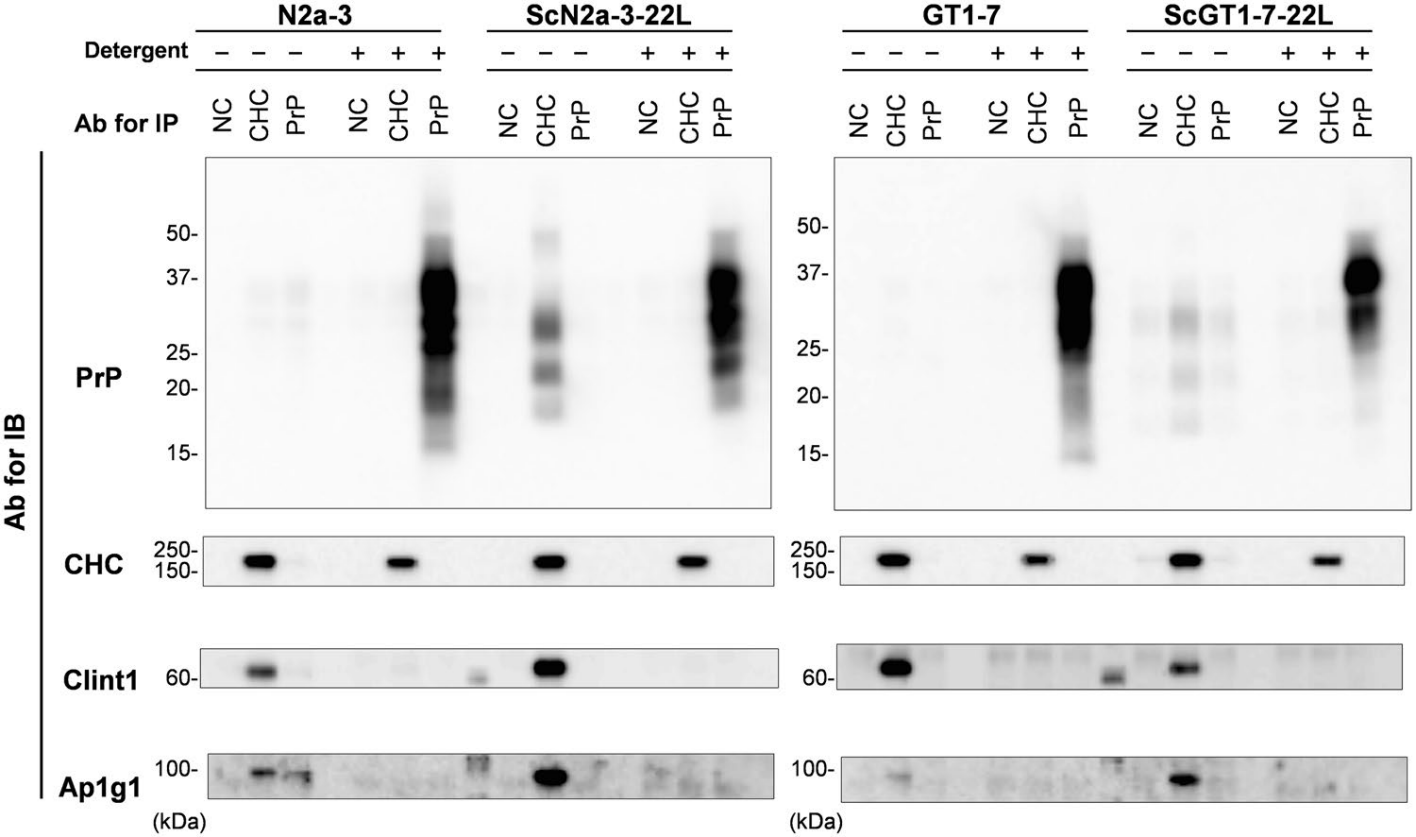
Detection of PrP after the immunoprecipitation of PNS with anti-CHC and anti-PrP antibodies. CHC and PrP were immunoprecipitated using anti-CHC antibodies, mAb 31C6, and control rabbit antibodies (NC) from the PNSs of N2a-3 and ScN2a-3-22L cells (left) and GT1-7 and ScGT1-7-22L cells (right), respectively, before and after the PNSs were treated with the detergent (detergent − and +). The precipitates were subjected to immunoblot analysis for PrP, CHC, Clint1, and Ap1g1. The figures show representative images of the immunoblot. The antibodies used for immunoprecipitation and immunoblotting are indicated on the top and the left of the figures, respectively. The cropped blots are shown in the figure, and full-length blots are presented in Supplementary Fig. S10.

In order to clarify the association of PrP^C^ or PrP^Sc^ with CCVs, we isolated CCVs from the PNS of uninfected or infected cells by immunoprecipitation using anti-CHC antibodies and analyzed PrP in the isolated fractions by immunoblotting (Fig. 4). CHC, Clint1, and Ap1g1 were detected in the fraction that was immunoprecipitated with anti-CHC antibodies from the PNS of N2a-3 and ScN2a-3-22L cells (Fig. 4, left, detergent -), indicating the successful isolation of CCVs. PrP signal was clearly detected in the CCVs from ScN2a-3-22L cells (Fig. 4, left, ScN2a-3-22L detergent -). However, only a slight signal of PrP^C^ was detected in CCVs from N2a-3 cells (Fig. 4, left, N2a-3 detergent -). The PrP signal detected in the CCVs from ScN2a-3-22L cells showed three major bands ranging from 35 to 20 kDa that were similar to the band pattern of N-terminal-truncated form of PrP^Sc^ reported in our previous study^32^. These results suggest that PrP in CCVs isolated from ScN2a-3-22L cells is PrP^Sc^, and that CCVs are associated with PrP^Sc^ rather than PrP^C^.

To further examine the manner of association of PrP^Sc^ with CCVs, the PNS was immunoprecipitated using anti-CHC and anti-PrP antibodies after it was treated with a detergent (Fig. 4, left, detergent +). None of the PrP, Clint1, or Ap1g1 signals were detected when detergent-treated PNSs from N2a-3 and ScN2a-3-22L cells were immunoprecipitated using anti-CHC antibodies. This result suggests that the detergent treatment disrupted the association between CCVs and PrP^Sc^. Although only a slight signal of PrP^C^ and no PrP signal were detected in PNSs from N2a-3 cells and ScN2a-3-22L cells that were immunoprecipitated with an anti-PrP antibody before the treatment with a detergent, clear PrP signals were detected in those from N2a-3 and ScN2a-3-22L cells after detergent treatment (Fig. 4, left, detergent −/+). Similar results were obtained when PNSs of GT1-7 and ScGT1-7-22L cells were immunoprecipitated using anti-CHC and anti-PrP antibodies before and after detergent treatment (Fig. 4, right). These results indicate that the anti-PrP antibody became accessible to PrP molecules under the condition that intracellular compartments, including CCVs, were disrupted. The inaccessibility of anti-PrP antibodies to PrP molecules associated with the intact intracellular compartments suggests that the PrP molecules are associated with not outside but inside of the intracellular compartments. Combined with the result that PrP was co-immunoprecipitated with CHC from the intact PNSs of ScN2a-3-22L and ScGT1-7-22L cells, these results at least indicate that PrP^Sc^ is present in CCVs in persistently prion-infected cells.

Next, we attempted to estimate the amount of PrP^Sc^ associated with the CCVs in ScN2a-3-22L cells (Supplementary Fig. S3). In order to analyze PrP^Sc^ in CCVs specifically involved in retrograde transport, the PNS from the ScN2a-3-22L cells was separated into 12 fractions by iodixanol density gradient centrifugation (Supplementary Fig. S3a), and the high density fractions (Fractions 6 and 7) that contained both CHC and Clint1 were used for immunoprecipitation with anti-CHC antibodies (Supplementary Fig. S3b). The amount of PrP co-immunoprecipitated with anti-CHC antibodies was nearly 1% of the total amount of PrP in the fractions (Supplementary Fig. S3c), suggesting that the estimated amount of PrP^Sc^ associated with CCVs was at the most less than 1% of the total PrP^Sc^ in the cells.

### Effects of Suppression of Clint1- and Ap1g1-Mediated Transport by CCVs on Intracellular Localization of PrP^Sc^

**Figure 5:**
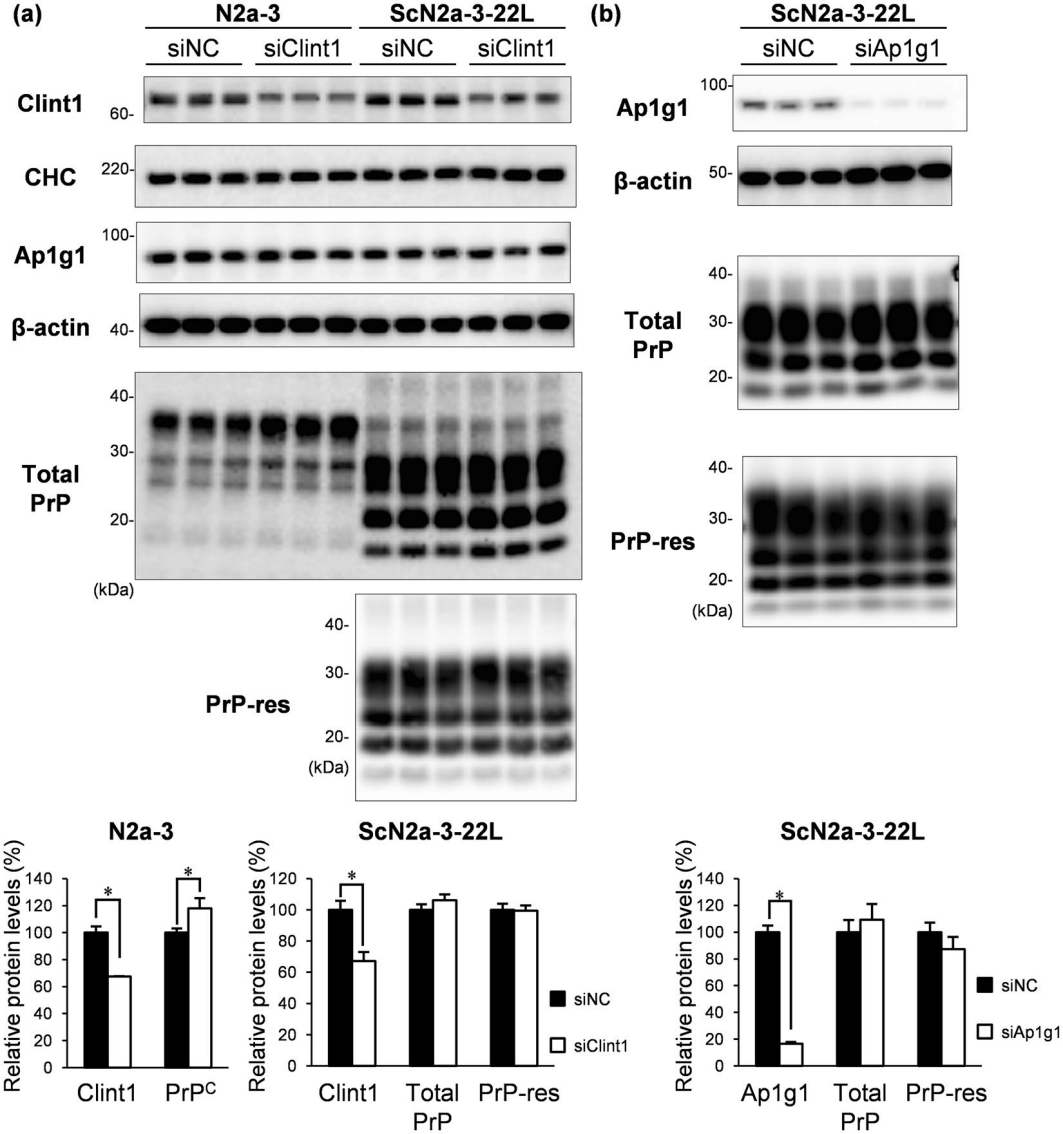
The effect of the knockdown of Clint1 and Ap1g1 on the amount of PrP in N2a-3 and ScN2a-3-22L cells. N2a-3 and ScN2a-3-22L cells were grown on 6-well plates. SiRNAs against Clint1 and Ap1g1 and negative control siRNA were transfected into the cells by lipofection. Seventy-two hours after the initiation of transfection, the cells were subjected to immunoblot analysis to monitor Clint1, CHC, Ap1g1, PrP^C^, total PrP, and PrP-res. β-actin was used as a loading control. The immunoblot images of the triplicate samples of N2a-3 and ScN2a-3-22L cells that were transfected with siRNA against Clint1 (siClint1) and negative control siRNA (siNC) are shown in (**a**). The immunoblot images of triplicated samples of ScN2a-3-22L cells that were transfected with siRNA against Ap1g1 (siAp1g1) and negative control siRNA (siNC) are shown in (**b**). The cropped blots are shown in the figure, and full-length blots are presented in Supplementary Figs S11 and S12. The graphs at the bottom show the levels of Clint1, Ap1g1, PrP^C^, total PrP, and PrP-res relative to the average of the control samples transfected with negative control siRNA. Mean and standard deviations (SDs) of triplicate samples are depicted. Asterisks indicate a significant decrease compared to the control (Student’s *t-*test, *p* < 0.05).

In order to examine whether CCVs are involved in the trafficking of PrP^Sc^, we performed gene silencing of Clint1 and Ap1g1 by transfection of siRNA into the cells. First, we analyzed the efficacy of siRNA knockdown and its effect on the amount of PrP^C^ and PrP^Sc^ in N2a-3 and ScN2a-3-22L cells (Fig. 5). Transfection of siRNA against Clint1 decreased the protein levels of Clint1 to 68% and 67% in N2a-3 and ScN2a-3-22L cells compared to the control siRNA transfected cells, respectively (Fig. 5a). The knockdown of Clint1 did not affect CHC and Ap1g1 levels in N2a-3 and ScN2a-3-22L cells, but it slightly but significantly increased the PrP^C^ level in N2a-3 cells (an 18% increase compared to the control). However, Clint1 knockdown did not affect total PrP and PrP-res levels in ScN2a-3-22L cells. Transfection of siRNA against Ap1g1 decreased the protein levels of Ap1g1 to 17% in ScN2a-3-22L cells compared to control cells, but it did not change the total PrP and PrP-res levels in ScN2a-3-22L cells (Fig. 5b).

**Figure 6:**
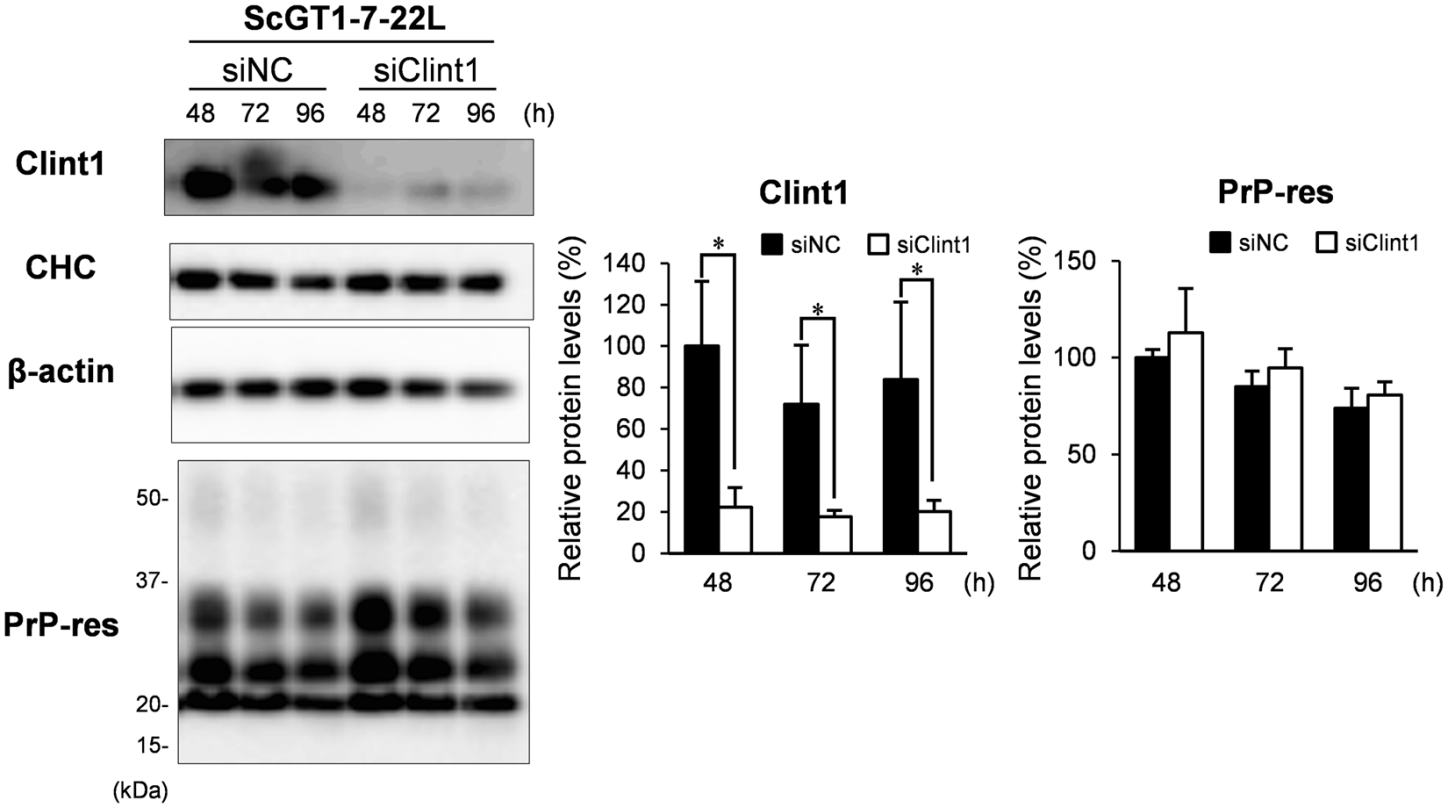
The effect of the knockdown of Clint1 on the amount of PrP in ScGT1-7-22L cells. SiRNAs against Clint1 (siClint1) and negative control siRNA (siNC) were transfected into ScGT1-7-22L cells by electroporation and plated on 12-well plates. After transfection, the cells were cultured for the indicated period and then subjected to immunoblotting to monitor Clint1, CHC, and PrP-res. β-actin was used as a loading control. The representative immunoblot images of three independent experiments are shown. The graphs on the right show the levels of Clint1 and PrP-res relative to the average of the control samples at 48 h after the transfection with negative control siRNA. The cropped blots are shown in the figure, and full-length blots are presented in Supplementary Fig. S13. Mean and SDs of three independent experiments are depicted. Asterisks indicate a significant decrease compared to the control (Student’s *t-*test, *p* < 0.05).

To confirm the effect of the knockdown of Clint1 on the amount of PrP^Sc^ in the other cell lines, we analyzed the amount of PrP-res in ScGT1-7-22L cells during the 48–96 h after transfection with siRNA against Clint1 (Fig. 6). The siRNA transfection decreased the protein level of Clint1 to 22% at 48 h after transfection, and the decreased levels of Clint1 were maintained for an additional 48 h. However, the levels of PrP-res did not change between the cells transfected with siRNA against Clint1 and those transfected with control siRNA during this period. These results suggest that the knockdown of Clint1 and Ap1g1 does not affect the amount of PrP^Sc^.

**Figure 7:**
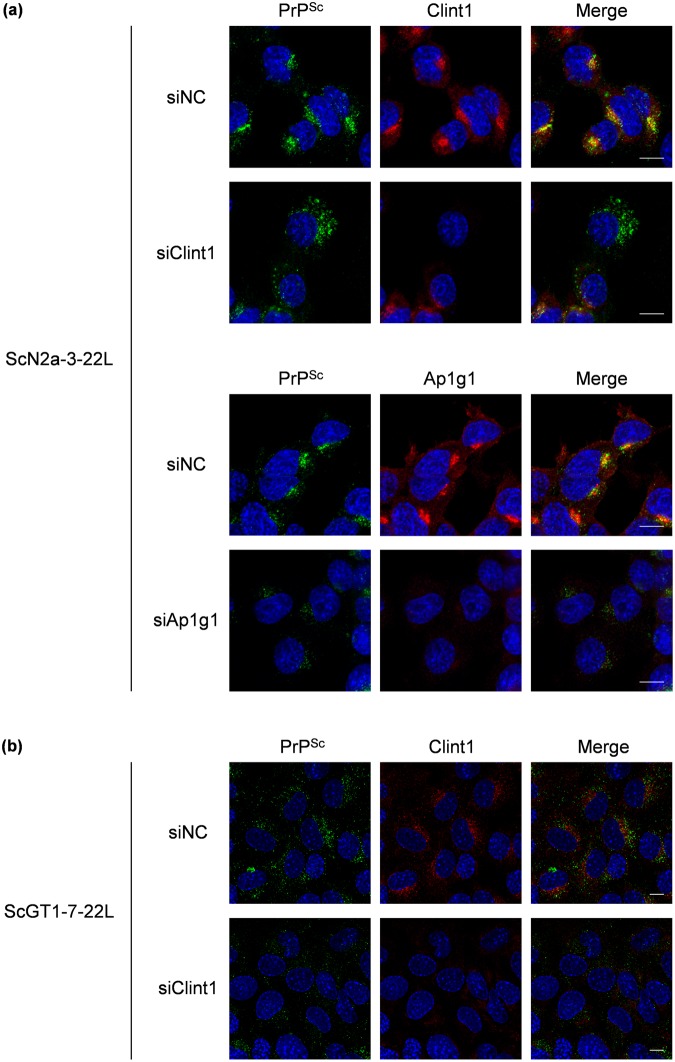
The effect of the knockdown of Clint1 and Ap1g1 on the intracellular localization of PrP^Sc^. SiRNAs against Clint1 (siClint1) and Ap1g1 (siAp1g1) and negative control siRNA (siNC) were transfected into ScN2a-3-22L cells by lipofection (**a**). Twenty-four hours after transfection, the cells were replated on Chambered Coverglass and cultured for 72 h. The cells were subjected to IFA to detect PrP^Sc^, Clint1, and Ap1g1. SiRNA against Clint1 and negative control siRNA were transfected into ScGT1-7-22L cells by electroporation (**b**). The cells were subjected to IFA to detect PrP^Sc^ and Clint1 96 h after the transfection. The cell nuclei were counterstained with DAPI. The panels show the merged images of PrP^Sc^ and nuclei (left), those of Clint1 or Ap1g1 and nuclei (center), and those of PrP^Sc^, Clint1, or Ap1g1 and nuclei (right). Scale bars: 10 µm.

Next, we analyzed the effect of the knockdown of Clint1 and Ap1g1 on the intracellular localization of PrP^Sc^ (Fig. 7 and Supplementary Fig. S4). The efficacy of the knockdown of Clint1 in ScN2a-3-22L cells was low (Fig. 5). Therefore, we analyzed only the cells that did not express Clint1 following transfection of siRNA against Clint1 into ScN2a-3-22L cells (Fig. 7a and Supplementary Fig. S4a). In the control siRNA transfected ScN2a-3-22L cells, most PrP^Sc^ signals clustered at the perinuclear region of the cells (Fig. 7a and Supplementary Fig. S4a, siNC). In contrast, the PrP^Sc^ that clustered at the perinuclear region disappeared, and large granular signals of PrP^Sc^ emerged at the peripheral region of the cells in which the expression of Clint1 was suppressed (Fig. 7a and Supplementary Fig. S4a, siClint1). The knockdown of Ap1g1 only reduced the strong PrP^Sc^ signals clustered at the region directly proximal to the nucleus (Fig. 7a and Supplementary Fig. S4a, siAp1g1). In the control siRNA transfected ScGT1-7-22L cells, PrP^Sc^ tended to localize around the nuclei of the cells (Fig. 7b and Supplementary Fig. S4b, siNC). Within the cells in which Clint1 expression was suppressed, the PrP^Sc^ around the nucleus disappeared and was instead dispersed throughout the cytoplasm (Fig. 7b and Supplementary Fig. S4b, siClint1). These results suggest that the retrograde transport by CCVs mediated by Clint1 or Ap1g1 is involved in the intracellular transport of PrP^Sc^.

**Figure 8:**
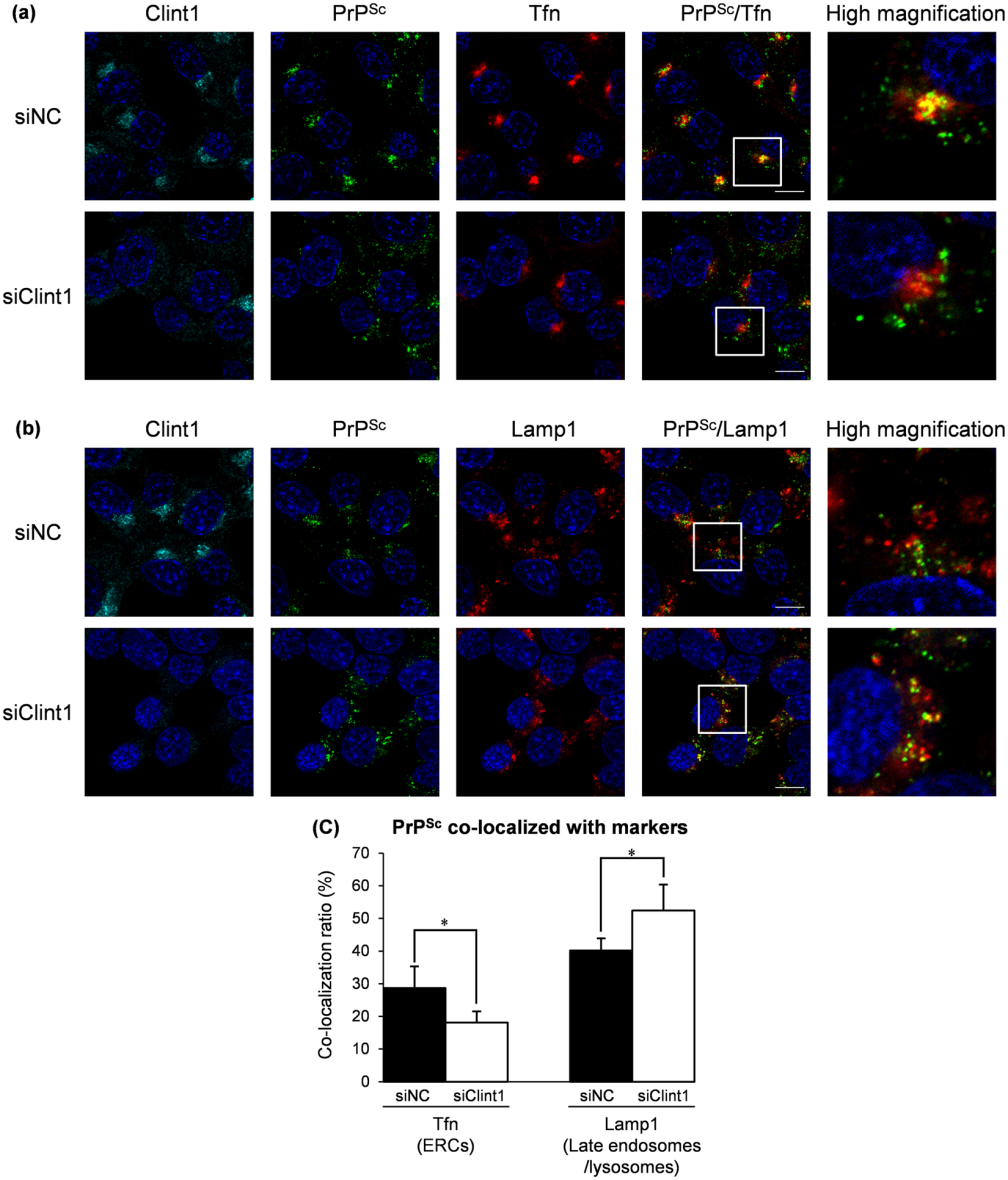
The effect of the knockdown of Clint1 on the co-localization of PrP^Sc^ with the markers for ERCs and late endosomes/lysosomes. ScN2a-3-22L cells were transfected with siRNA against Clint1 (siClint1) or control siRNA (siNC) similarly as shown in Fig. 7. In order to label ERCs, the cells were incubated with fluorescent-labeled Tfn for 15 min and then fixed with PFA (**a**). To label the late endosomes and lysosomes, the cultured cells were stained for Lamp1 after fixation with PFA (**b**). The fixed cells were subjected to IFA for PrP^Sc^ and Clint1. The cell nuclei were counterstained with DAPI. Z-series of the images were acquired at 0.8 µm steps from the top to the bottom of the cells in the five view fields. The panel shows the representative images of the signals of Clint1 (cyan), PrP^Sc^ (green), marker molecules (red), and nuclei (blue) in cells transfected with the siRNA indicated on the left. The merged images of Clint1 and nuclei, those of PrP^Sc^ and nuclei, and those of Lamp1 and nuclei are shown in the leftmost, the second left, and the third left columns, respectively. The merged images of PrP^Sc^, marker molecules, and nuclei are shown on the second right column. The rightmost column displays the higher-magnification images of the boxed regions in the second right column. The ratios of PrP^Sc^ signals co-localized with exogenously introduced Tfn or the ratio of PrP^Sc^ signals co-localized with Lamp1 relative to the total signals of PrP^Sc^ are shown in the graph (**c**). The mean and SDs of five view fields are depicted. Asterisks indicate a significant increase or decrease compared to the control (Student’s *t-*test, *p* < 0.05). Scale bars: 10 μm.

In order to characterize the change in the intracellular localization of PrP^Sc^ when retrograde transport by CCVs was inhibited, we performed a co-localization analysis of PrP^Sc^ with exogenously introduced Tfn, an ERC marker, and Lamp1, a marker of late endosomes/lysosomes (Fig. 8). In ScN2a-3-22L cells in which the expression of Clint1 was suppressed, the strong signals of PrP^Sc^ co-localized with Tfn at perinuclear regions disappeared (Fig. 8a). In contrast, the large granular signals of PrP^Sc^ clearly co-localized with Lamp1 appeared at the peripheral region of the cells (Fig. 8b). The co-localization ratio of PrP^Sc^ with Tfn was significantly decreased (from 28.7% to 18.1%), and that of PrP^Sc^ with Lamp1 was significantly increased (from 40.2% to 52.4%) by the knockdown of Clint1 (Fig. 8c). These results suggest that the suppression of Clint1 induces the redistribution of PrP^Sc^ from Tfn-positive ERCs to Lamp1-positive late endosomes/lysosomes.

## Discussion

In the present study, we showed that a portion of PrP^Sc^ in persistently prion-infected cells is intracellularly transported by CCVs. CCVs are known to be involved in multiple membrane trafficking pathways of cells, such as endocytosis from the plasma membrane, intracellular transport between endosomes and TGN, and transport from early endosomes to lysosomes. For each trafficking pathway, the cell uses specific types of clathrin adaptor proteins^33^. CCVs that act with Ap1 and Clint1 specifically mediate the transport between endosomes and TGN^28,29,34,35^. Previous reports showed that PrP^Sc^ was detected at clathrin-coated pits of the plasma membrane of neuroblastoma cells infected with prions^11^, and that PrP^Sc^ was detected at the abnormal clathrin-coated structures in the brains of prion-infected animals^36^, suggesting that some PrP^Sc^ on the cell surface is internalized into cells by clathrin-dependent endocytosis. In contrast to these reports, our present data showed that PrP^Sc^ was co-localized with CHC, Ap1g1, and Clint1 at perinuclear regions rather than peripheral regions near the plasma membrane in persistently prion-infected cells (Figs 1 and 2). In addition, suppression of Ap1g1 and Clint1 redistributed PrP^Sc^ from the perinuclear region, including ERCs, to peripheral regions, such as late endosomes and lysosomes (Figs 7 and 8). Taken together, the CCV-associated transport of PrP^Sc^ shown by our present data is not considered to be clathrin-dependent endocytosis or transport from early endosomes to lysosomes, but it is specific to the retrograde transport from endosomes to TGN. Although the estimated amount of PrP^Sc^ associated with the CCVs was less than 1% of the total amount of PrP^Sc^ in the cells (Supplementary Fig. S3), it is meaningful that the biological significance of the association between PrP^Sc^ and CCVs was shown in the present study. Regarding the transport of cargo protein by CCVs, the clathrin coating is known to occur during the vesicle budding^37^. After fission of the CCV from the original membrane compartment, clathrin is removed from CCV and the uncoated vesicle is transported to the target membrane compartment^38^. Considering that the formation of CCVs occurs only during the limited process in the membrane trafficking, it is reasonable that only small amount of PrP^Sc^ is associated with CCVs. Furthermore, we showed that gene silencing of Clint1 and Ap1g1 altered the intracellular localization of PrP^Sc^ (Figs 7 and 8), suggesting that the CCVs is actually involved in the transport of PrP^Sc^ even though the amount of PrP^Sc^ associated with the CCVs is less than 1%. To our knowledge, this is the first time within the field of cell biology and prion disease research that the involvement of CCVs in the intracellular transport of PrP^Sc^ is clearly shown.

The transport from endosomes to TGN by CCVs is considered to be one of the pathways mediating the recycling of molecules, such as membrane proteins, lipids, and lysosomal enzymes, by transferring the molecules from endosomal compartments to secretory compartments^26^. Although CCVs are known to be involved in the retrograde transport from early endosomes to the TGN, it is suggested that there are two pathways for cargo proteins to reach the TGN: direct transport from early endosomes to the TGN^28,39^ and indirect transport from early endosomes to the TGN via ERCs^29^. It is reported that Clint1 and Ap1 mediate the transport of CTxB from ERCs to the TGN^29^. Meanwhile, Clint1 is reported to be involved in the direct transport of STxB from early endosomes to the TGN^28,39^. Considering these reports, the Clint1-mediated transport by CCVs might be employed in different endosomes, such as early endosomes and ERCs, depending on the cargo proteins. Here we showed that the distribution of PrP^Sc^ was not increased but decreased at ERCs by the suppression of Clint1 (Fig. 8). This result suggests that the Clint1-mediated transport by CCVs is involved in the transport from early endosomes to ERCs in the case of PrP^Sc^.

In the prion-infected cells in which Clint1 was suppressed, the PrP^Sc^ increased at late endosomes/lysosomes (Fig. 8). Previously, we suggested that a portion of PrP^Sc^ is circulating between the perinuclear and peripheral regions, including the plasma membrane via ERCs, but another portion of PrP^Sc^ is directed to the compartments of the endolysosomal pathway^13^. We also reported that the redistribution of PrP^Sc^ from the endocytic-recycling pathway to the endolysosomal pathway by disturbing membrane trafficking machineries with chlorpromazine or U18666A facilitates the degradation of PrP^Sc^ in lysosomes^20^. The inhibition of retrograde transport from early endosomes to the TGN by suppression of Clint1 or Ap1g1 was expected to result in the increase of PrP^Sc^ in early endosomes, with this facilitating the transport of PrP^Sc^ from early endosomes to the endolysosomal pathway for degradation. However, the amount of PrP^Sc^ remained unchanged (Figs 5 and 6), suggesting that the inhibition of Clint1- and Ap1g1-mediated transport via CCVs is not sufficient to affect the generation or degradation of PrP^Sc^. ERCs and MVBs/late endosomes are considered to be the sites where the conversion of PrP^C^ to PrP^Sc^ occurs^12,17^. We also reported that endosomal compartments within both the endocytic-recycling and the endolysosomal pathways are important for the generation of PrP^Sc^ ^18^. Taking this into consideration, the redistribution of PrP^Sc^ from ERCs to late endosomes might not affect the total generation of PrP^Sc^ unless it is actively transported into lysosomes for degradation.

Alternatively, the machinery involved in the recycling of PrP^Sc^ via ERCs may not be limited to the retrograde transport from early endosomes to the TGN by CCVs, as there may be multiple pathways involved. We showed that PrP^Sc^ is co-localized with Rab9 but barely with Tip47 (Figs 1 and 2). This may suggest that Rab9/Tip47 complex is not involved in the transport of PrP^Sc^. Our previous and present data showed that PrP^Sc^ in persistently prion-infected cells is co-localized with Rab11a^13^ (Supplementary Fig. S2), which is involved in the transport from ERCs to the plasma membrane^40^, suggesting the possible role of this pathway in the recycling of PrP^Sc^, as suggested by a previous report^19^. In addition, PrP^Sc^ is co-localized with the components of the retromer complex in ScN2a-3-22L and ScGT1-7-22L cells (Figs 1 and 2). Although clathrin and retromer complexes are considered to coordinate retrograde transport at different endosomal subdomains^31^, their precise relationship still remains unclear^41^. At the subcellular localization level of ScGT1-7-22L cells, components of the retromer complex were present with PrP^Sc^ within low density fractions, which contained small amounts of Clint1 and Ap1g1 but large amounts of CHC (Fig. 3d). This may suggest that the retromer complex acts with clathrin for PrP^Sc^ transport independently of Clint1 and Ap1g1. Taken together with the report that the retromer-mediated transport passes through ERCs^42^, this retromer-mediated transport is another candidate pathway for the recycling of PrP^Sc^ via ERCs. This idea is supported by previous reports stating that the retromer complex is involved in the intracellular transport of PrP^Sc^ in persistently prion-infected cells^17^, as well as in the very early stage after prion infection of cells^19^. Multiple pathways, such as retromer-mediated transport and Rab11a-mediated recycling, may compensate the recycling of PrP^Sc^ via ERCs even when Clint1- or Ap1g1-mediated transport is inhibited. Furthermore, these multiple trafficking pathways involving ERCs may complement each other for the continuous generation of PrP^Sc^ in persistently prion-infected cells.

Although we demonstrated that some PrP^Sc^ is intracellularly transported by CCVs in Neuro2a and GT1-7 cells persistently infected with prions, the pathological significance of this finding within neurons should be carefully considered. Neurons are one of the most polarized cells and are characterized by specialized morphologies; they are clearly compartmentalized into pre- and post-synaptic regions, synapse, dendrite, soma, and axon^43^. The mechanisms of membrane trafficking and protein sorting are variable between these distinct neuronal compartments in order to maintain precise functioning at each compartment with correct functional molecular composition. In fact, axons differ from soma and dendrites, which are referred to as somatodendritic compartments, in the machineries for secretory and endocytic pathways as shown by the differences in the sorting signals, motor proteins, and organelles required for trafficking^44^. Moreover, the distribution of endosomes is not uniform between axons and dendrites; EEA1-positive early endosomes and MVBs are more distributed within somatodendritic compartments than within axons^45,46^, whereas recycling endosomes are spread almost evenly throughout these compartments^44^. Considering these polarized membrane trafficking systems in neurons, it could be difficult to simply apply our findings to *in vivo* phenomena. When prions are orally inoculated into animals, they are thought to reach their initial CNS target sites by spreading in a retrograde direction along efferent fibers of both sympathetic and parasympathetic nerves^47^. During the trafficking from distal to proximal regions of sympathetic and parasympathetic nerves, PrP^Sc^ must be transported from axonal compartments to somatodendritic compartments in the neurons. Concerning the intraneuronal transport related to clathrin, CCVs and Ap1 are considered to be involved in the transport from axonal compartments to somatodendritic compartments^48^. We previously reported that a majority of PrP^Sc^ is present at the plasma membrane of primary cultured cortical neurons, but a relatively small portion of PrP^Sc^ is confirmed to exist in the cytoplasm of somatodendritic compartments^32^. Considering these data, the intracellular transport of PrP^Sc^ via CCVs could contribute to the transport of PrP^Sc^ from the axonal to the somatodendritic compartments of neurons, which might be involved in the neuroinvasion of prions from the peripheral nervous systems to the CNS. Of course, more detailed analyses of PrP^Sc^ trafficking in neurons, peripheral nervous systems, and the CNS in prion-infected animals are required. These extensive analyses on the machineries involved in the intracellular trafficking of PrP^Sc^ will contribute to the clarification of their pathological roles in prion-infected animals.

## Methods

### Antibodies

Anti-PrP mouse monoclonal antibodies (mAbs) 31C6 and 132 were used to detect PrP^49^. The other commercially available primary and secondary antibodies used for immunoblot analysis and IFA are listed in Supplementary Table S1. Antibodies were used for IFA and immunoblotting at the dilution recommended by the manufacturers. Alexa Fluor 555-conjugated Tfn (Thermo Fisher Scientific) was used as a marker for ERCs. Fab fragments of mAb 31C6 genetically conjugated with human placental alkaline phosphatase (31C6Fab-PLAP, A. S. and M. H., unpublished) were used for the direct detection of PrP in immunoblot analyses. Alexa Fluor 488-conjugated mAb 132, which was prepared as described previously^50^, was used for the direct immunostaining of PrP^Sc^ in IFA.

### Cell Culture

The following cell lines were used: N2a-3 cells, a subclone of the mouse neuroblastoma cell line Neuro2a^51^; the hypothalamic neuronal cell lines GT1, GT1-7^52^; N2a-3 cells persistently infected with the 22 L prion strain (ScN2a-3-22L); and GT1-7 cells persistently infected with the 22 L prion strain (ScGT1-7-22L)^13^.

### Immunoblot Analysis

The preparation of samples to monitor PrP-res or other molecules, SDS-PAGE, and immunoblotting were performed as previously described^13,51^.

### IFA

IFA, including PrP^Sc^-specific staining, was carried out as described previously^18,20^. The specificity of PrP^Sc^ staining was confirmed by staining of uninfected N2a-3 cells and GT1-7 cells as negative control, and by staining of N2a-3 cells and GT1-7 cells that were inoculated with microsome fraction prepared from 22 L prion strain-infected mouse brains^32^ as positive control (Supplementary Fig. S5). Confocal fluorescent images were acquired with Objective Plan-Apochromat 63x lens (numerical aperture: 1.40) on a Zeiss LSM700 inverted microscope and ZEN 2009 software (Zeiss). The pinhole size was adjusted to 47 μm. Z-series of the images were taken at every 0.8 μm from the top to the bottom of the cells in the target area. About 12–20 and 5–15 z-stack images per view field were acquired for the co-localization analysis on ScN2a-3-22L cells and ScGT1-7-22L cells, respectively.

### Co-localization Statistics

Quantitative co-localization analysis of PrP^Sc^ with the components of CCVs, those of the retromer complex, Tfn or Lamp1 was performed as previously described^18^. The co-localization ratio representing a percentage of the voxels of PrP^Sc^ signals co-localized with the signals of each marker relative to the total voxels of PrP^Sc^ signals was quantified using the Coloc module with Imaris software (Bitplane).

### Subcellular Fractionation by Iodixanol Density Gradient Centrifugation

Subcellular fractionation using iodixanol solution was carried out according to the OptiPrep manufacturer’s instructions (60% iodixanol; Axis-Shield) with some modifications. N2a-3 and ScN2a-3-22L cells were plated onto four Nunclon Delta Treated Square BioAssay Dishes (Thermo Fisher Scientific) at a 1: 4 ratio and cultured for four days. GT1-7 and ScGT1-7-22L cells were plated onto six T175 flasks (Thermo Fisher Scientific) at a 1: 3 ratio and cultured for six days. The cells were harvested in PBS with a cell scraper and combined into a new tube for each cell line. The cells were pelleted by centrifugation at 400 × *g* at room temperature (RT) for 5 min, and the supernatant was removed. The cells were washed with 6 mL of homogenization buffer (HB) containing 250 mM sucrose, 10 mM Tris-HCl (pH = 7.5), and 1 mM EDTA. After pelleting, the cells were incubated in 6 mL of HB containing protease inhibitors: 5 μM E-64 protease inhibitor, 2 μg/mL leupeptin, 5 μM bestatin hydrochloride, and 1 μg/mL pepstatin A (Sigma-Aldrich), at 4 °C for 60 min, and homogenized by 50 strokes of Dounce Homogenizer. After the removal of the crude plasma membrane fraction and nuclear fraction by centrifugation at 2,000 × *g* at 4 °C for 15 min, the PNS was obtained. In order to remove the cytosolic fraction, the PNS was pelleted by ultracentrifugation at 40,000 rpm at 4 °C for 60 min using S55A rotor (Hitachi). The samples resuspended in 1 mL of HB were loaded on top of 0–40% continuous iodixanol gradients, which were prepared by mixing 5 mL of HB and 5 mL of 40% iodixanol solution by Gradient Master according to the manufacturer’s instructions (Biocomp Instruments), and the samples were centrifuged at 40,000 rpm at 4 °C for 18 h using an SW41Ti rotor (Beckman). After centrifugation, 0.9 mL of the fractions was collected from the top to the bottom of each sample to yield 12 fractions. The densities of the fractions were determined by measuring the optical density at 350 nm. The fractions were diluted with four times the volume of HB and centrifuged at 45,000 rpm at 4 °C for 60 min using an S80AT3 rotor (Hitachi). The pellets were lysed with a lysis buffer containing 0.5% Triton X-100, 0.5% sodium deoxycholate, 10 mM Tris-HCl (pH = 7.5), 150 mM NaCl, and 5 mM EDTA. Protein concentrations of the lysates were measured with a DC Protein Assay (Bio-Rad), and the relative amounts of protein in each fraction to the total amount of protein in the 12 fractions were determined. The lysates were mixed with equivalent volumes of 2x SDS sample buffer and subjected to immunoblot analysis. In order to monitor PrP-res in each fraction, the protein concentration of each lysate was adjusted to 0.8 mg/mL using the cell lysate of N2a-3 cells before PK treatment. PK treatment and the preparation of the SDS sample were performed as previously described^51^.

### Isolation of CCVs by Immunoprecipitation

The PNS or subcellular fractions as prepared above were used. PNS equivalent to 0.5 mg of protein was resuspended in 0.9 mL of HB containing the above-mentioned proteinase inhibitors. For detergent treatment, the PNS was incubated with 0.5% Triton X-100 and 0.5% sodium deoxycholate at 4 °C for 1 h. Immunoprecipitation using anti-CHC antibodies, mAb 31C6 and control rabbit antibodies, was carried out according to the manufacturer’s instructions of Dynabeads Protein G (Thermo Fisher Scientific). Briefly, 5 μg of antibodies was bound to 50 μL of Dynabeads Protein G. The Dynabeads-bound antibodies were incubated at 4 °C for over 4 h with 1 mL of the blocking solution: HB containing 1% I-Block (Thermo Fisher Scientific) and 5% fetal bovine serum (FBS) for detergent-untreated samples or HB containing 1% I-Block, 10% Blocking Regent-N101 (NOF Corporation), and 3% Tween 20 for detergent-treated samples. The PNS treated with or without a detergent was incubated with the Dynabeads-bound antibodies in 1 mL of HB containing a 10% blocking solution at 4 °C overnight. After the supernatant was collected separately, the Dynabeads-bound samples were eluted in 50 μL of SDS sample buffer. The immunoprecipitation step was repeated again for the supernatant when subcellular fractions were used for the immunoprecipitation as starting material.

### Gene Silencing

The siRNAs against Clint1 (siGENOME SMARTpool; M-063671-00), Ap1g1 (ON-TARGETplus SMARTpool; L-057175-01), and control siRNA (Nontargeting siRNA Pool #1; D-001206-13) used for gene silencing were obtained from Dharmacon. Transfection of siRNA into N2a-3 and ScN2a-3-22L cells was performed by lipofection using Lipofectamine 2000 (Thermo Fisher Scientific) according to the manufacturer’s instructions. The cells were seeded onto 24-well and 6-well plates at a density of 4 × 10^4^ and 2 × 10^5^ cells per well, respectively, and cultured in Dulbecco’s modified Eagle’s medium (DMEM; ICN Biomedicals) containing 10% FBS and MEM nonessential amino acids (NEAA; Thermo Fisher Scientific) at 37 °C for 24 h before transfection. Twenty picomole and 100 pmol of siRNAs were diluted in 50 and 125 μL of Opti-MEM, respectively, and mixed with an equivalent volume of Opti-MEM containing 2.8% Lipofectamine 2000. The cells cultured on the 24-well and 6-well plates were incubated with 20 and 100 pmol of siRNAs in Opti-MEM at 37 °C for 24 h, respectively. The cells on the 24-well plates were resuspended in 1.5 mL of DMEM containing 10% FBS and NEAA, and 0.5 mL of the cell suspension was transferred to each well of 8-well Lab-Tek II Chambered Coverglass (Thermo Fisher Scientific). The cells cultured at 37 °C for 72 h were subjected to IFA. The cells incubated with siRNAs on 6-well plates for 24 h were subsequently cultured in fresh DMEM at 37 °C for 48 h and then analyzed using immunoblotting.

Transfection of siRNAs into GT1-7 and ScGT1-7-22L cells was performed via electroporation using the Neon Transfection System (Thermo Fisher Scientific) according to the manufacturer’s instructions. Briefly, 2.4 × 10^5^ cells were resuspended in 100 μL of resuspension buffer R containing 100 pmol of siRNA and pulsed with a single pulse of 1,350 V for 30 ms. The cells were resuspended in DMEM containing 5% FBS and 5% horse serum and split into 12-well plates and 8-well Lab-Tek II Chambered Coverglass at a density of 8 × 10^4^ and 2 × 10^4^ cells per well, respectively. After incubation at 37 °C for 24 h, the medium was replaced with fresh DMEM, and the cells were subsequently cultured for 24–72 h followed by immunoblot analysis and IFA.

## Electronic supplementary material

Supplementary Information

## Data Availability

All data generated or analyzed during this study are included in this published article (and its Supplementary Information files).
