# Supplementary material for: Retrograde Transport by Clathrin-Coated Vesicles is Involved in Intracellular Transport of PrPSc in Persistently Prion-Infected Cells
Source: Sci Rep. 2018 Aug 16;8:12241. doi: 10.1038/s41598-018-30775-1 (PMC6095914; doi:10.1038/s41598-018-30775-1)
Supplement: Supplementary file 1 — Supplementary Information [file 41598_2018_30775_MOESM1_ESM.pdf]

Retrograde Transport by Clathrin-Coated Vesicles Is Involved in Intracellular Transport of PrP^{Sc} in Persistently Prion-Infected Cells

Takeshi Yamasaki[1], Akio Suzuki[1], Rie Hasebe[1], and Motohiro Horiuchi[1,2]

[1]Laboratory of Veterinary Hygiene, Faculty of Veterinary Medicine, Graduate School of Infectious Diseases, Hokkaido University, Kita 18, Nishi 9, Kita-ku, Sapporo 060-0818, Japan

[2]Global Station for Zoonosis Control. Global Institute for Collaborative Research and Education, Hokkaido University, Kita 20, Nishi 10, Kita-ku, Sapporo 001-0020, Japan

Supplementary Information

Supplementary Table S1: Sources of commercially available antibodies.

	Antibodies	Company	Code number
Primary antibodies	Anti-clathrin heavy chain polyclonal antibodies	Abcam	Ab21679
	Anti-Clint1 rabbit polyclonal antibodies	Proteintech	10470-1-AP
	Anti-Ap1g1 rabbit polyclonal antibodies	Sigma-Aldrich	AV51923
	Anti-Snx1 rabbit polyclonal antibodies	Proteintech	10304-1-AP
	Anti-Vps26 rabbit polyclonal antibodies	abcam	Ab23892
	Anti-Vps29 goat polyclonal antibodies	abcam	Ab10160
	Anti-Vps35 goat polyclonal antibodies	Everest BIOTECH	EB06268
	Anti-Rab9 rabbit polyclonal antibodies	Proteintech	11420-1-AP
	Anti-Tip47 rabbit polyclonal antibodies	Lifespan BioScience	LS-C18110
	Anti-Rab4a rabbit polyclonal antibodies	Proteintech	10347-1-AP
	Anti-Rab5 rabbit polyclonal antibodies	Cell Signaling Technology	#2143
	Anti-Rab11a rabbit monoclonal antibody clone D4F5	Cell Signaling Technology	#5589
	Anti-Rab7 rabbit monoclonal antibody clone D95F2	Cell Signaling Technology	#9367
	Anti-Lamp1 rat monoclonal antibody clone 1D4B	Merck	MABC39
	Anti-cathepsin D rabbit monoclonal antibody clone EPR3057Y	Abcam	ab75852
	Anti-Tgn38 rabbit polyclonal antibodies	Abcam	ab16059
	Anti-giantin rabbit poly clonal antibodies	Covance	PRB-114C
	Anti-β-actin mouse monoclonal antibody clone AC15	Sigma-Aldrich	A5316
Secondary antibodies	Alexa Fluor 488-conjugated goat $F(ab')_2$ fragment anti-mouse IgG	Thermo Fisher Scientific	A-11017
	Alexa Fluor 488-conjugated donkey IgG anti-mouse IgG	Thermo Fisher Scientific	A-21202
	Alexa Fluor 555-conjugated goat IgG anti-rat IgG	Thermo Fisher Scientific	A-21434
	Alexa Fluor 555-conjugated goat $F(ab')_2$ fragment anti-rabbit IgG	Thermo Fisher Scientific	A-21430
	Alexa Fluor 647-conjugated goat $F(ab')_2$ fragment anti-rabbit IgG	Thermo Fisher Scientific	A-21246
	Alexa Fluor 555-conjugated donkey IgG anti-goat IgG	Thermo Fisher Scientific	A-21432
	Anti-Mouse IgG, HRP-Linked F(ab')2 Fragment Sheep	GE Healthcare	NA9310
	Anti-Rabbit IgG, HRP-Linked F(ab')2 Fragment Donkey	GE Healthcare	NA9340
	Rabbit Anti-Goat IgG Antibody, HRP conjugate	Merck	AP106P

Supplementary Figure S1

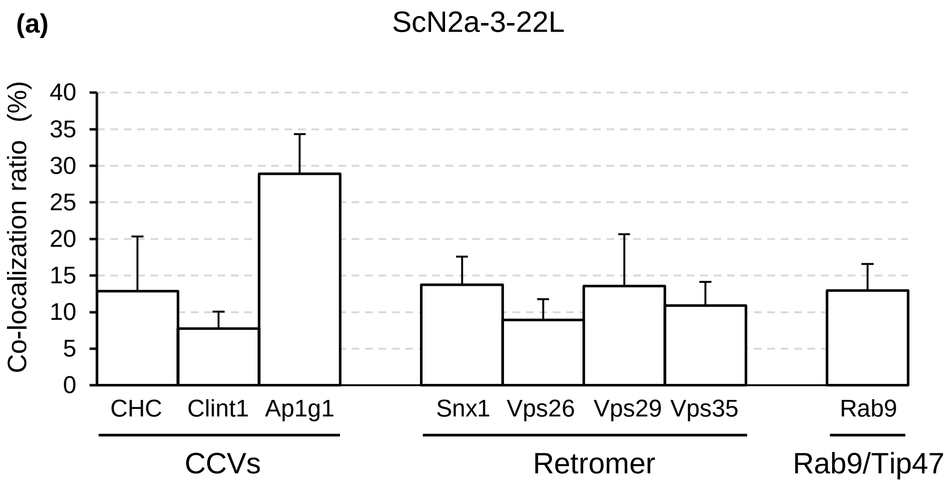

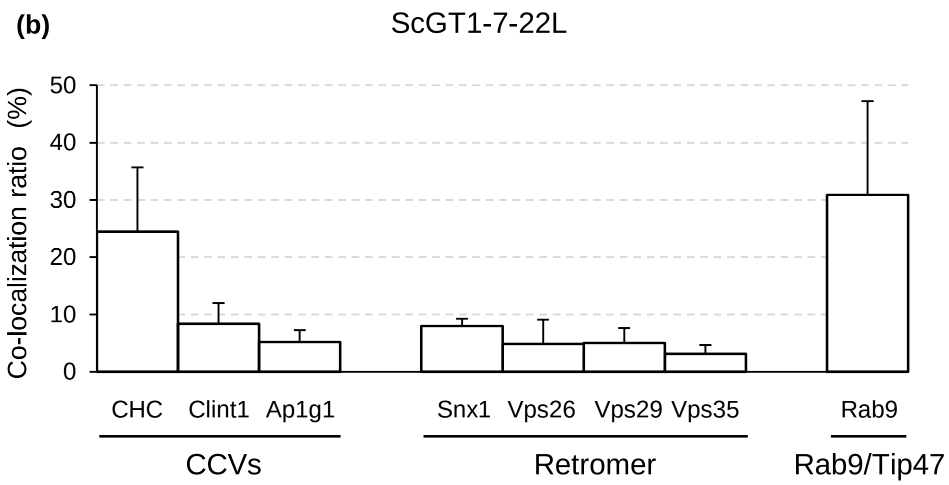

Supplemental Figure S1: Co-localization statistics. Ratios of PrP^{Sc} signals co-localized with each component molecule of clathrin coated vesicles (CCVs); CHC, Clint1, and Ap1g1, ratios of PrP^{Sc} signals co-localized with each component molecule of the retromer complex; Snx1, Vps26, Vps29, and Vps35, and ratio of PrP^{Sc} signals co-localized with Rab9 relative to the total signals of PrP^{Sc} in ScN2a-3-22L cells (a) and ScGT1-7-22L cells (b) were quantified as described in section “Methods”. The mean and SDs of the value acquired in six view fields are depicted.

Consistent with the images in Figs 1 and 2, PrP^{Sc} in ScN2a-3-22L cells and ScGT1-7-22L cells were partially co-localized with the component molecules of CCVs, those of the retromer complex, and Rab9.

Supplementary Figure S2

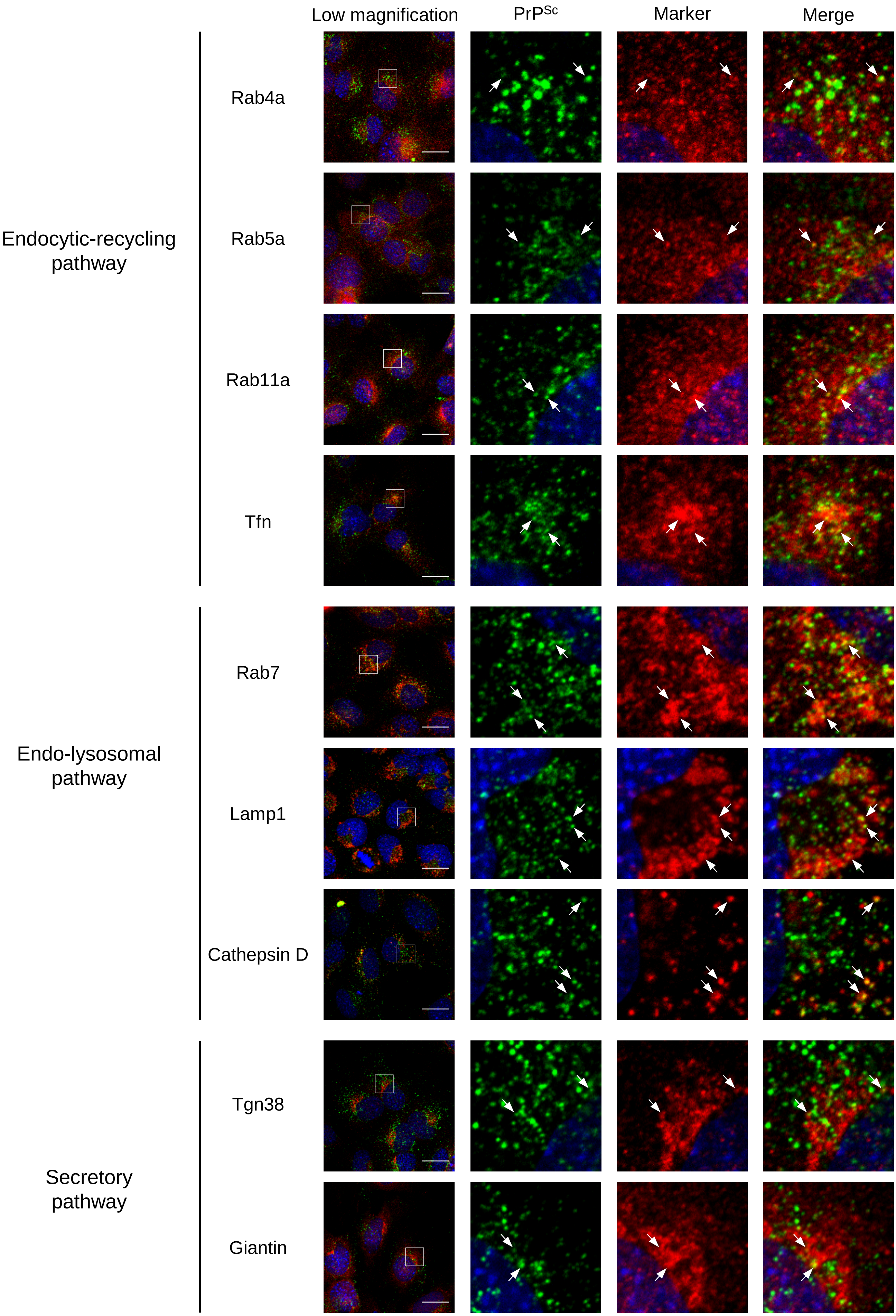

Supplemental Figure S2: Intracellular localization of PrP^{Sc} in ScGT1-7-22L cells. ScGT1-7-22L cells grown on Chambered Coverglass for 96 h were subjected to IFA to monitor PrP^{Sc} and organelle marker molecules. The leftmost column shows merged images of PrP^{Sc} (green), the organelle marker molecules indicated on the left (red), and nuclei (blue). The other three images in the row are the corresponding high-magnification images of the boxed region for the merged image of PrP^{Sc} and nuclei (second left), for the merged image of the marker molecules and nuclei (second right), and for the merged images of PrP^{Sc}, the marker molecules, and nuclei (rightmost). The arrows indicate representative examples of areas where PrP^{Sc} and the corresponding organelle marker molecules are co-localized. The co-localization areas were defined as pixels that were positive for both PrP^{Sc} signals and signals of the corresponding marker molecules. Scale bars: 10 µm.

PrP^{Sc} was partially co-localized with Rab4a, a marker of early endosomes including rapid endocytic recycling endosomes; Rab5a, a marker of early endosomes; Rab11a, a marker of recycling endosomes; and exogenously introduced Tfn, a marker of endocytic-recycling compartments, at perinuclear region. In contrast, PrP^{Sc} was partially co-localized with Rab7, a marker of late endosomes; Lamp1, a marker of late endosomes and lysosomes; and cathepsin D, a marker of lysosomes, at relatively peripheral region of the cells. In contrast to the co-localization of PrP^{Sc} with these markers of organelles in endocytic-recycling pathway and endolysosomal pathway, only a small portion of PrP^{Sc} was co-localized with Tgn38, a marker of the TGN, and giantin, a marker of the cis/medial-Golgi, which are organelles in secretory pathway.

Supplementary Figure S3

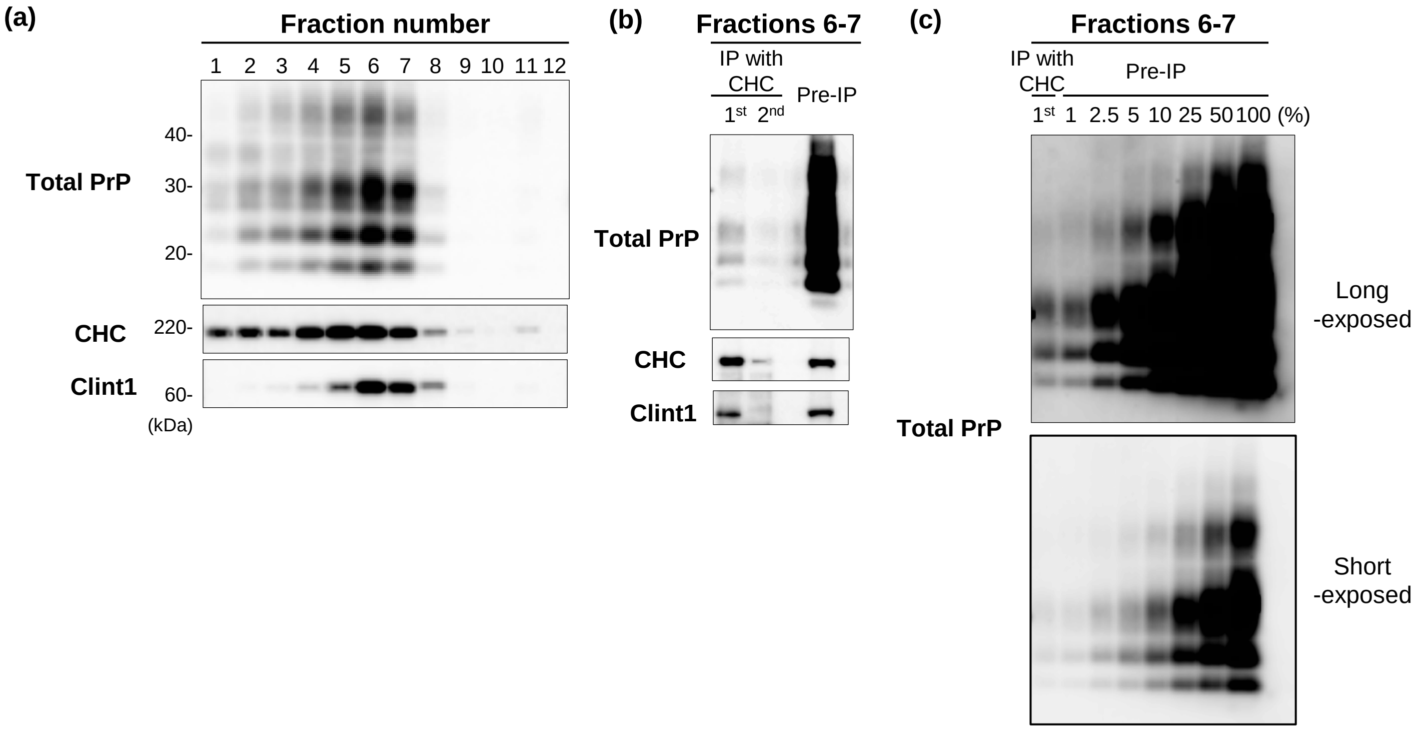

Supplemental Figure S3: The amount of PrP molecules associated with CCVs. PNSs prepared from ScN2a-3-22L cells were separated into 12 fractions by iodixanol gradient centrifugation. Fractions 6 and 7 that contained both CHC and Clint1 were combined and used for immunoprecipitation with anti-CHC antibodies. Following immunoprecipitation, the supernatant was subsequently used again for immunoprecipitation with anti-CHC antibodies to completely collect CCVs from the fraction. The 12 separated fractions and the immunoprecipitates were subjected to immunoblot analysis for PrP, CHC, and Clint1. The immunoblot image of the 12 separated fractions, the image of precipitates of the first and second immunoprecipitation (IP with CHC 1st and 2nd) with input fractions 6-7 (Pre-IP), and the image of precipitates of the first immunoprecipitation (IP with CHC 1st) with serially diluted input fractions 6-7 (Fraction 6-7, Pre-IP, 1%–100%) are shown in (a), (b), and (c), respectively. The long-exposed image (top) and short-exposed image (bottom) of the same immunoblot are shown in (c). The cropped blots are shown in the figure, and full-length blots are presented in Supplementary Fig. S14.

The amount of PrP co-immunoprecipitated with anti-CHC antibodies by the first immunoprecipitation was nearly 1% of the total amount of PrP in the fractions. PrP^{Sc} present in the PNS was estimated to be the portion of PrP^{Sc} that was present in the entire cell fraction because the crude plasma membrane fraction was removed when the PNS was prepared. Taken together, the estimated amount of PrP^{Sc} associated with CCVs was at the most less than 1% of the total PrP^{Sc} in the cells.

Supplementary Figure S4

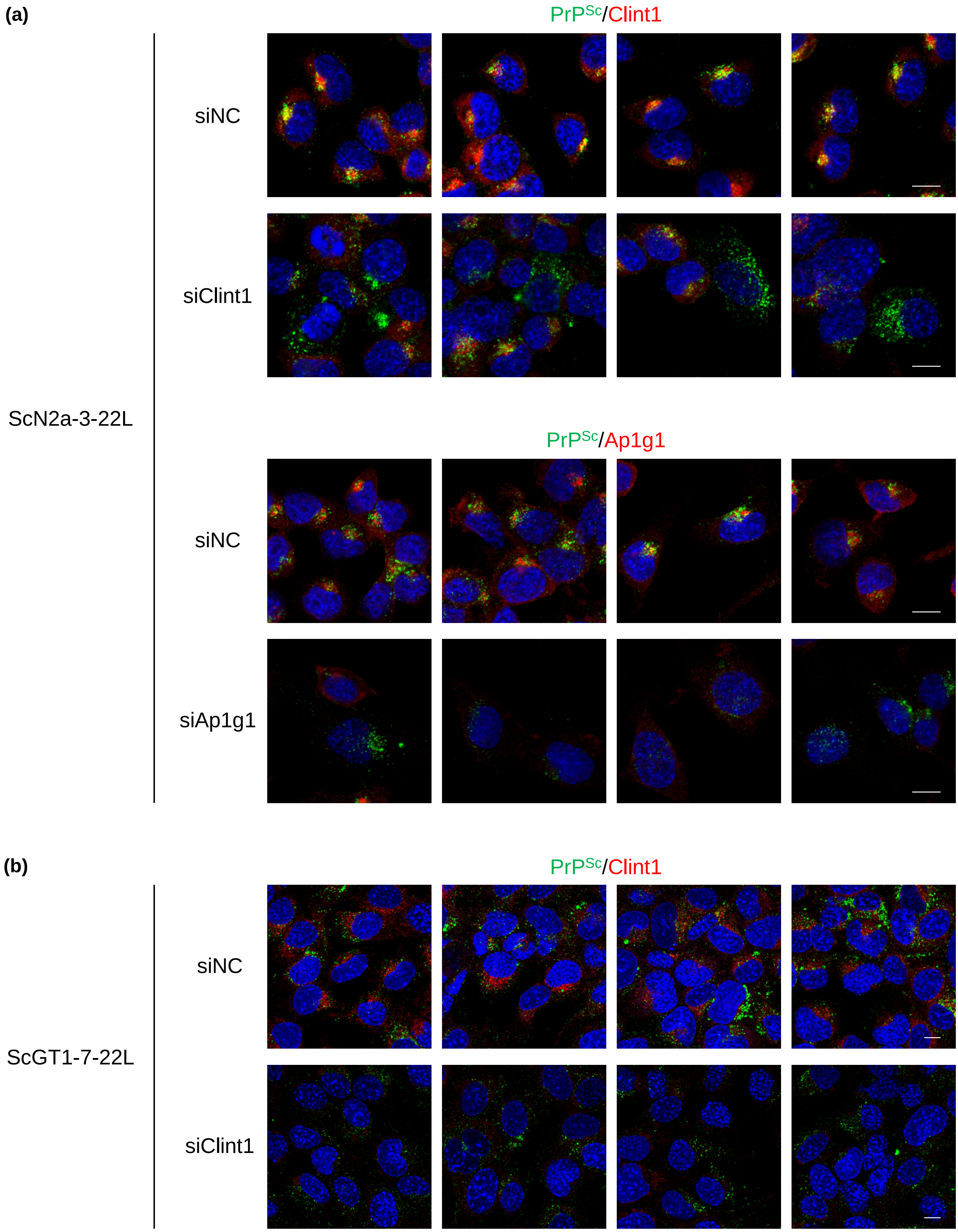

Supplemental Figure S4: The effect of the knockdown of Clint1 and Ap1g1 on the intracellular localization of PrP^{Sc}. SiRNAs against Clint1 (siClint1) and Ap1g1 (siAp1g1) and negative control siRNA (siNC) were transfected into ScN2a-3-22L cells (a) and ScGT1-7-22L cells (b), and cultured under the same condition as described in Figure 7. The cells were subjected to IFA to detect PrP^{Sc}, Clint1, and Ap1g1. The cell nuclei were counterstained with DAPI. The panels show the merged images of PrP^{Sc} (green), Clint1 or Ap1g1 (red), and nuclei (blue). Scale bars: 10 µm.

The alteration in PrP^{Sc} distribution by knockdown of Clint1 and Ap1g1 was observed in different view fields, which is consistent with the data shown in Figure 7.

Supplementary Figure S5

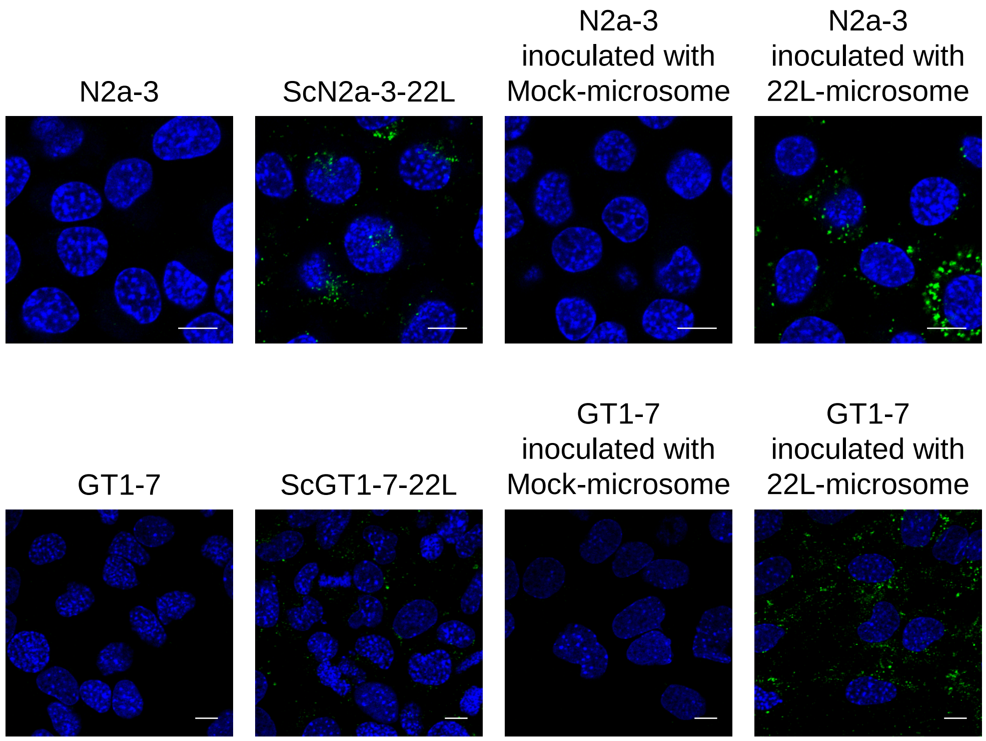

Supplemental Figure S5: Specific staining of PrP^{Sc}. N2a-3 cells and ScN2a-3-22L cells were grown on the 8 well-Chambered Coverglass for 72 h. GT1-7 cells and ScGT1-7-22L cells were grown on the 8 well-Chambered Coverglass for 96 h. Microsome fractions were prepared from un-infected mouse brains (mock-microsome) or prepared from 22L-prion strain-infected mouse brains (22L-microsome). N2a-3 cells and GT1-7 cells were incubated with the mock-microsome or 22L-microsome equivalent to 10 ng of total protein for 6 h. Un-infected N2a-3 cells and GT1-7 cells, persistently prion-infected ScN2a-3-22L cells and ScGT1-7-22L cells, N2a-3 cells and GT1-7 cells inoculated with the mock-microsome or 22L-microsomes were subjected to the staining of PrP^{Sc} with mAb 132. The cell nuclei were counterstained with DAPI. The panels show the merged images of PrP^{Sc} (green) and nuclei (blue). Scale bars: 10 µm.

PrP^{Sc} signals were detected in N2a-3 cells and GT1-7 cells that were inoculated with 22L-microsome, but not detected in un-infected N2a-3 cells and GT1-7 cells, and in N2a-3 cells and GT1-7 cells that were inoculated with mock-microsome. This result indicates that 22L-microsome-derived PrP^{Sc} was specifically detected. Under these condition, the PrP^{Sc} signals were detected both in persistently prion-infected ScN2a-3-22L cells and ScGT1-7-22L cells, confirming the specificity of PrP^{Sc} staining in these cells.

Supplementary Figure S6

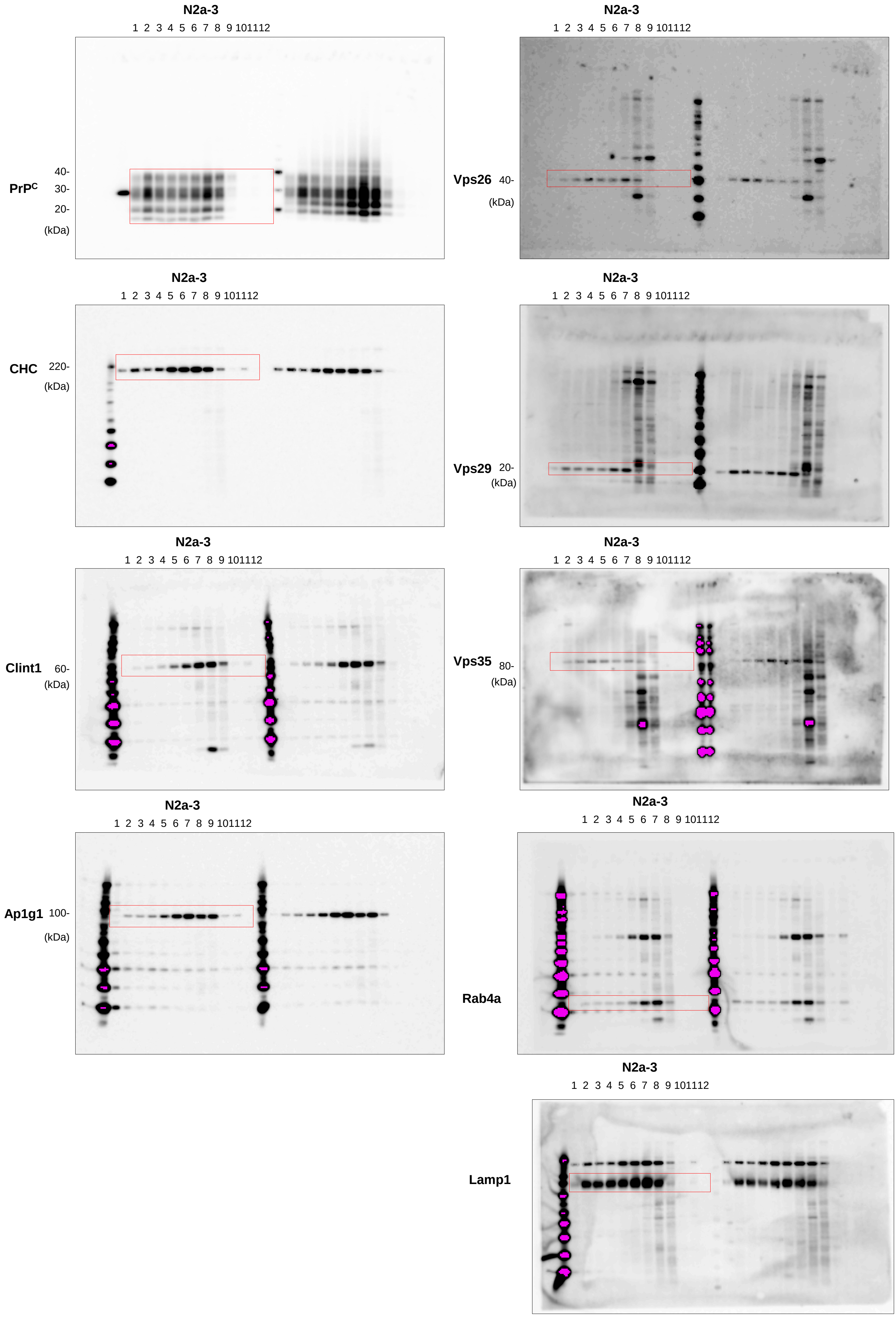

Supplemental Figure S6: Full-length blots of Figure 3a. Red-boxed regions are shown in Fig. 3a.

Supplementary Figure S7

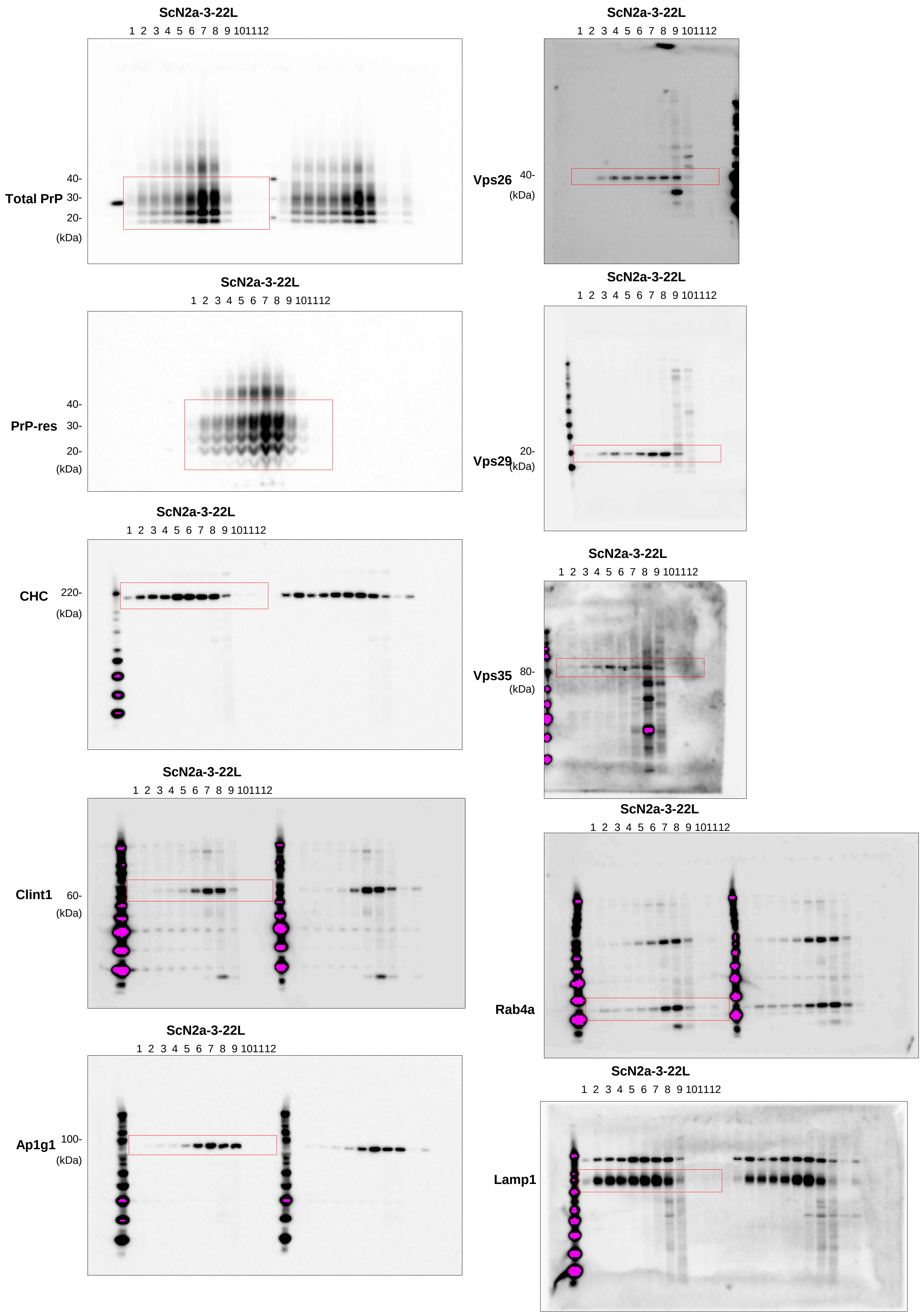

Supplemental Figure S7: Full-length blots of Figure 3b. Red-boxed regions are shown in Fig. 3b.

Supplementary Figure S8

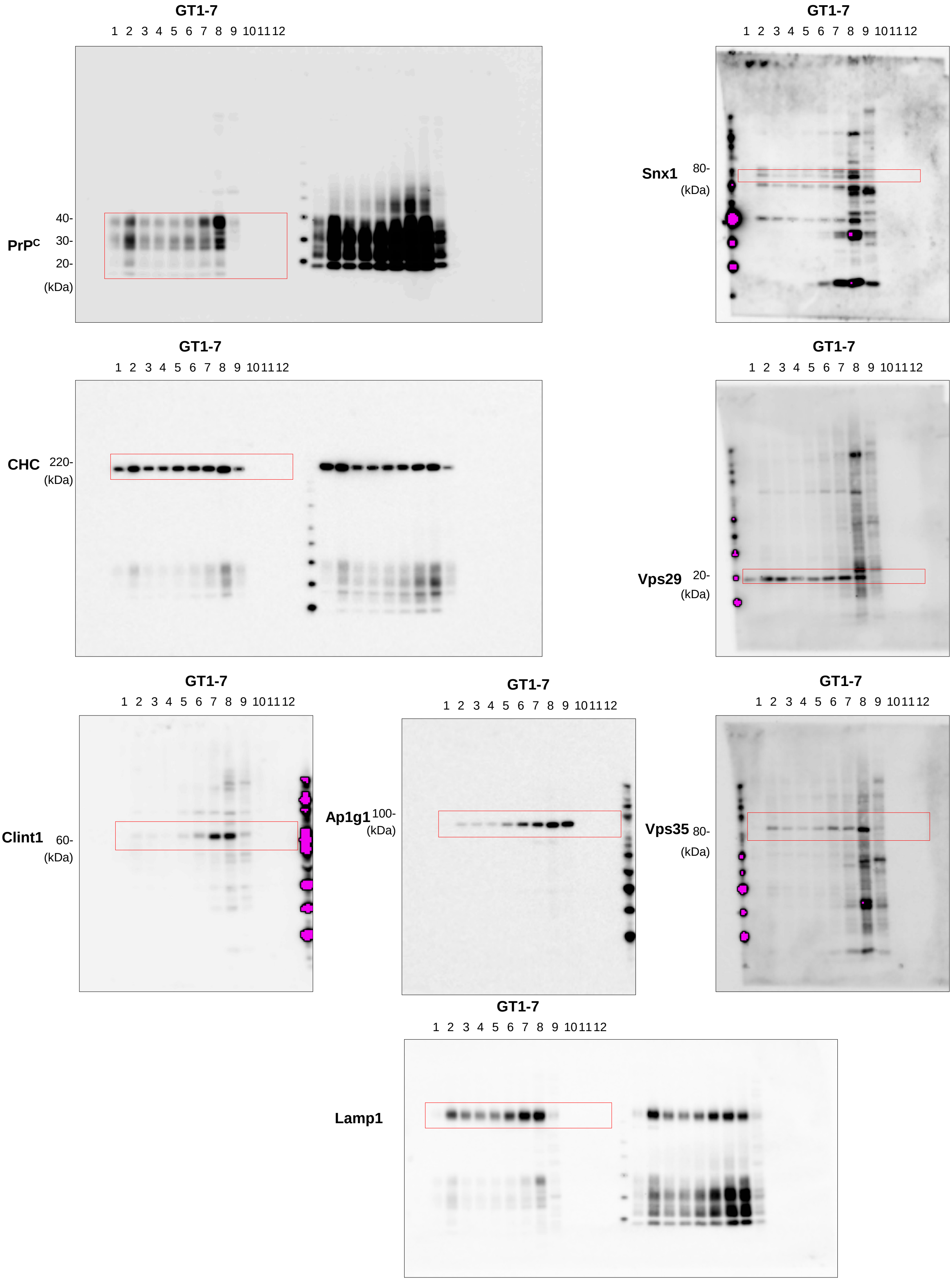

Supplemental Figure S8: Full-length blots of Figure 3c. Red-boxed regions are shown in Fig. 3c.

Supplementary Figure S9

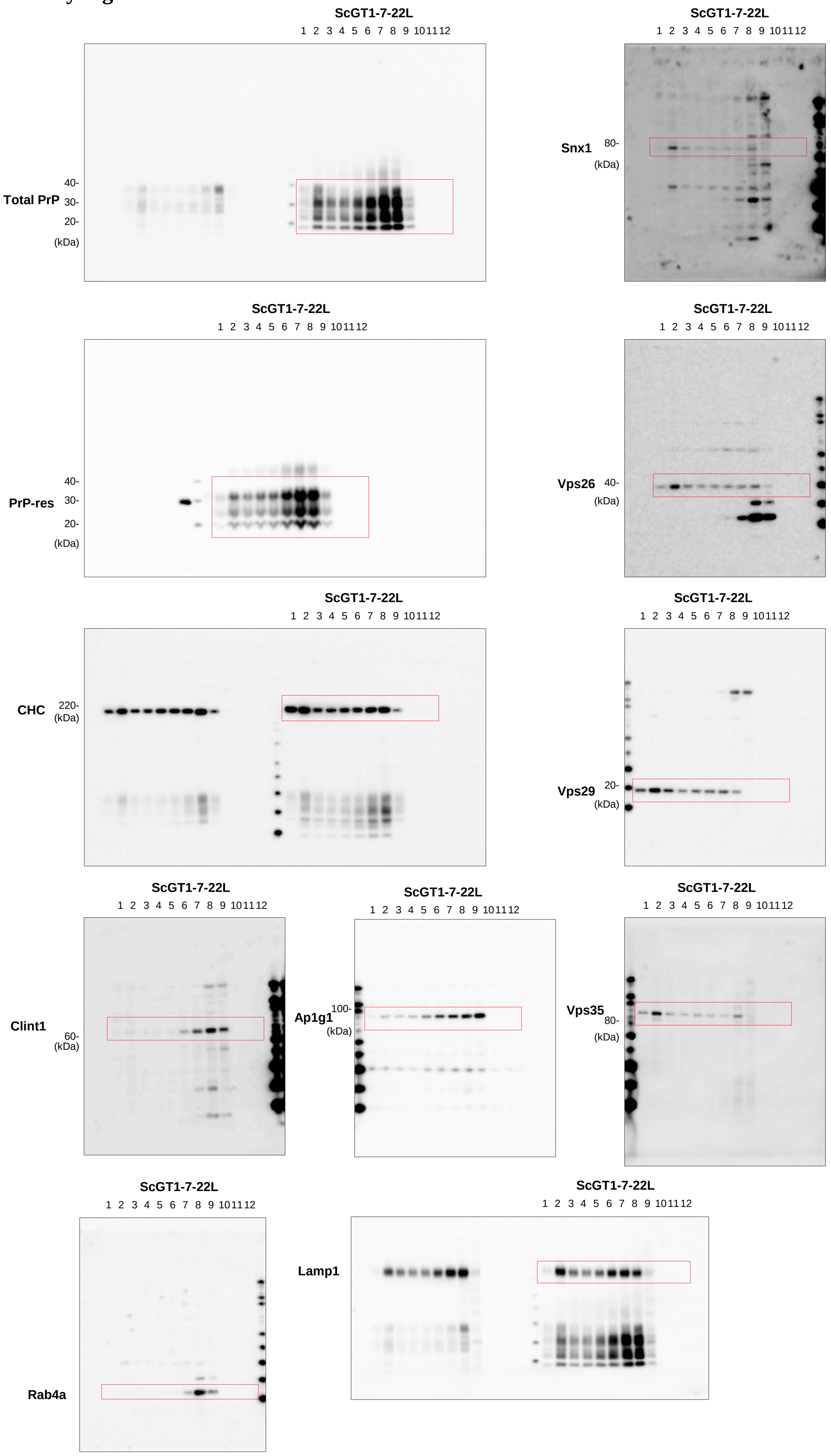

Supplemental Figure S9: Full-length blots of Figure 3d. Red-boxed regions are shown in Fig. 3d.

Supplementary Figure S10

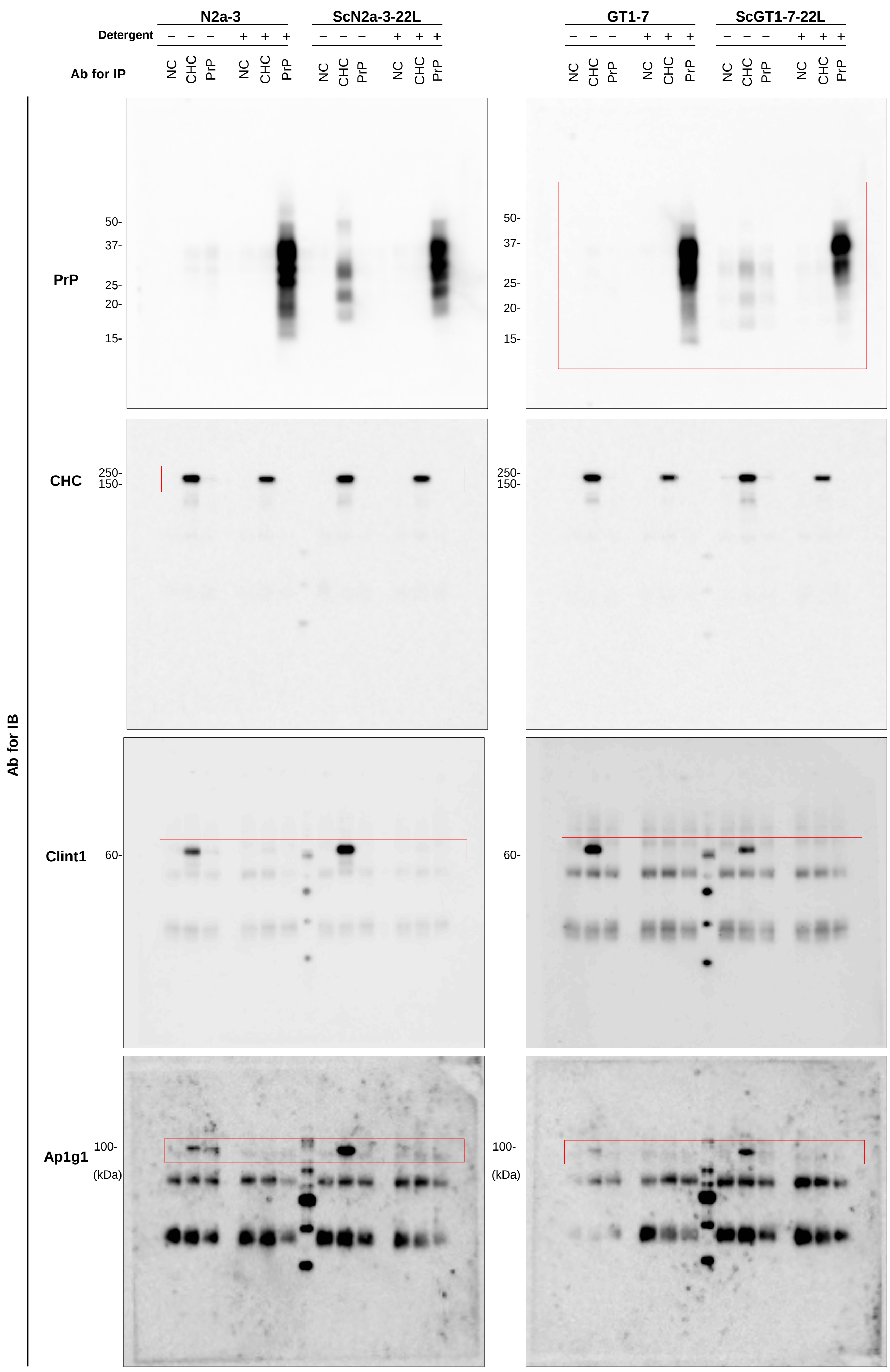

Supplemental Figure S10: Full-length blots of Figure 4. Red-boxed regions are shown in Fig. 4.

Supplementary Figure S11

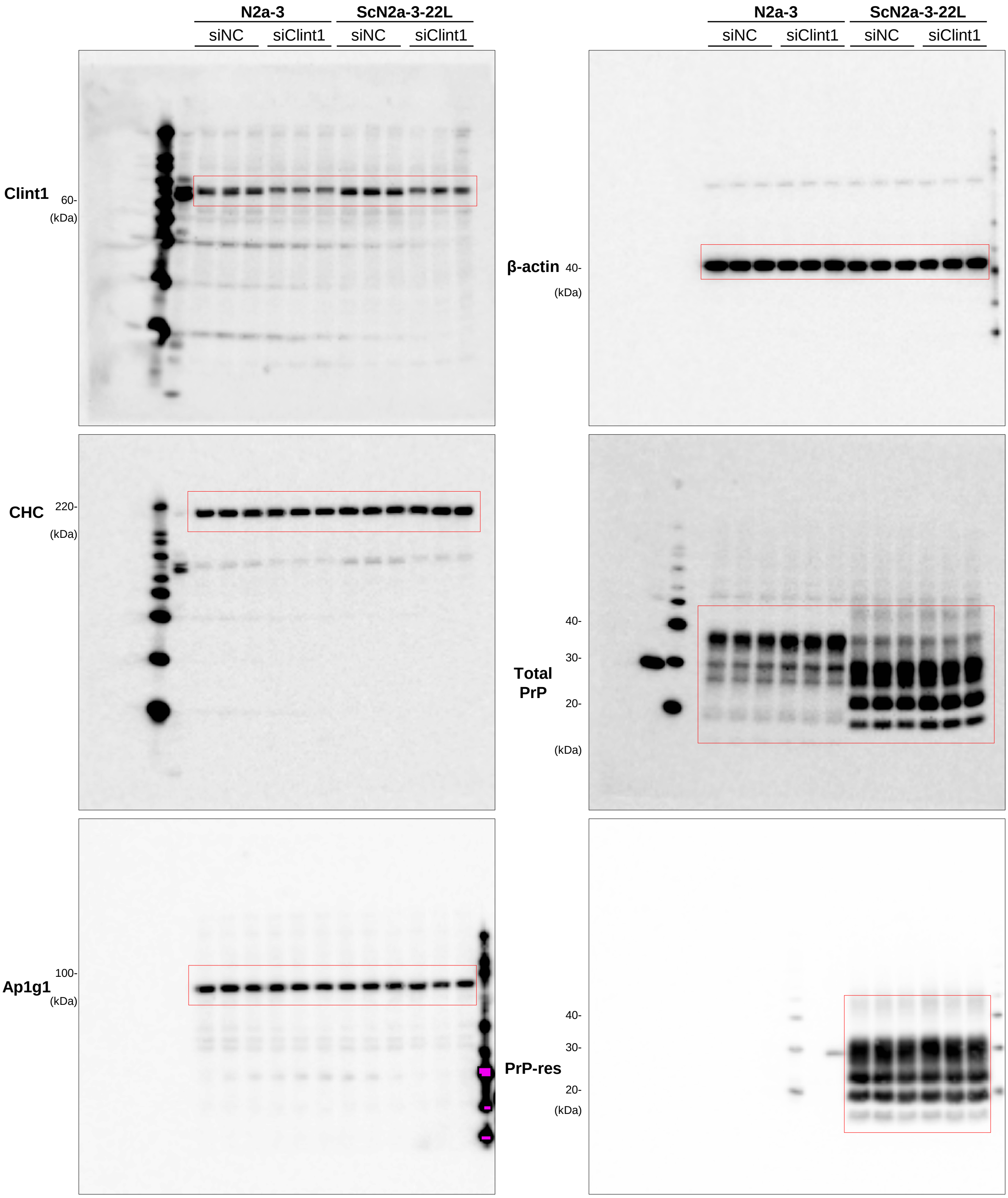

Supplemental Figure S11: Full-length blots of Figure 5a. Red-boxed regions are shown in Fig. 5a.

Supplementary Figure S12

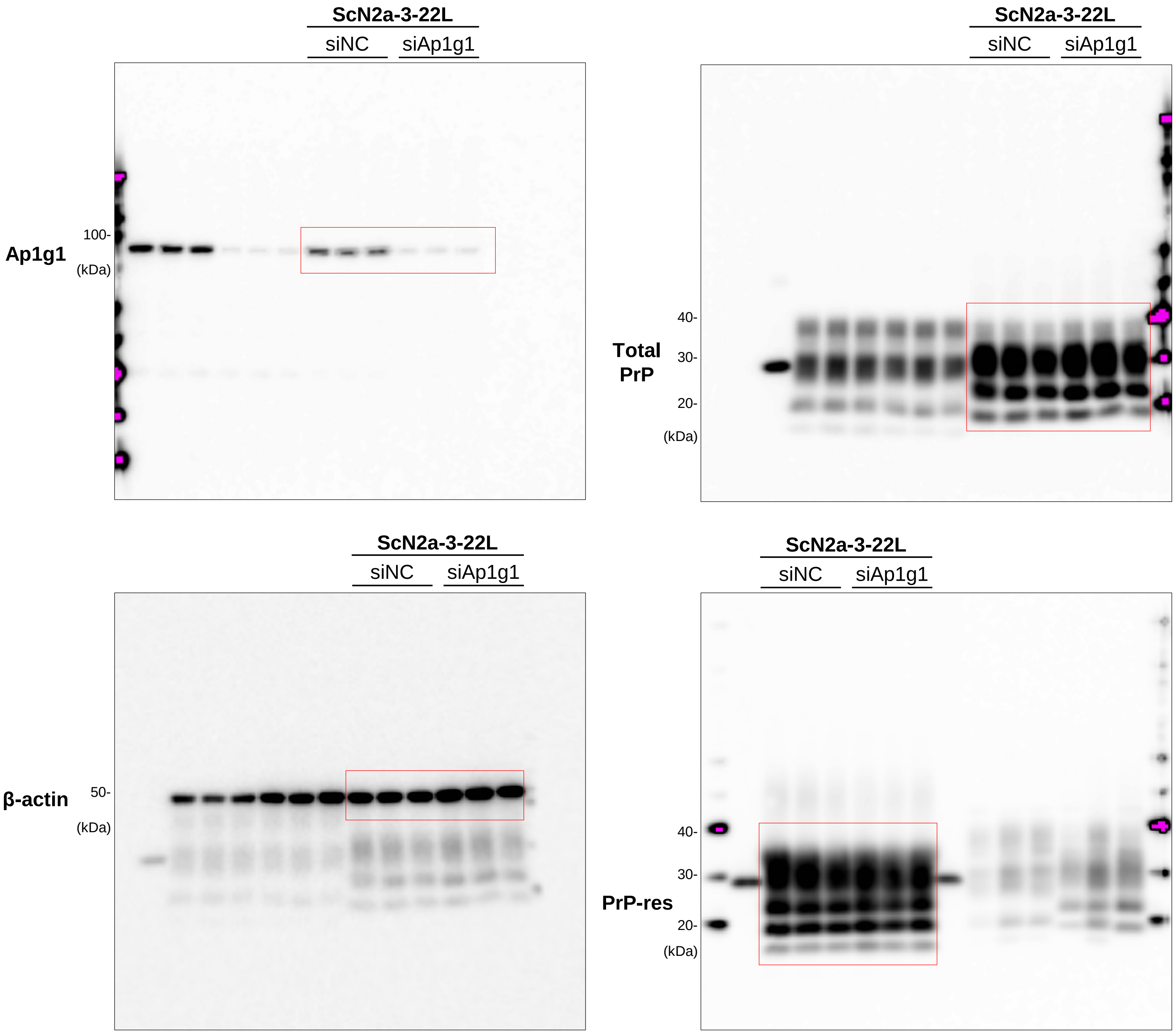

Supplemental Figure S12: Full-length blots of Figure 5b. Red-boxed regions are shown in Fig. 5b.

Supplementary Figure S13

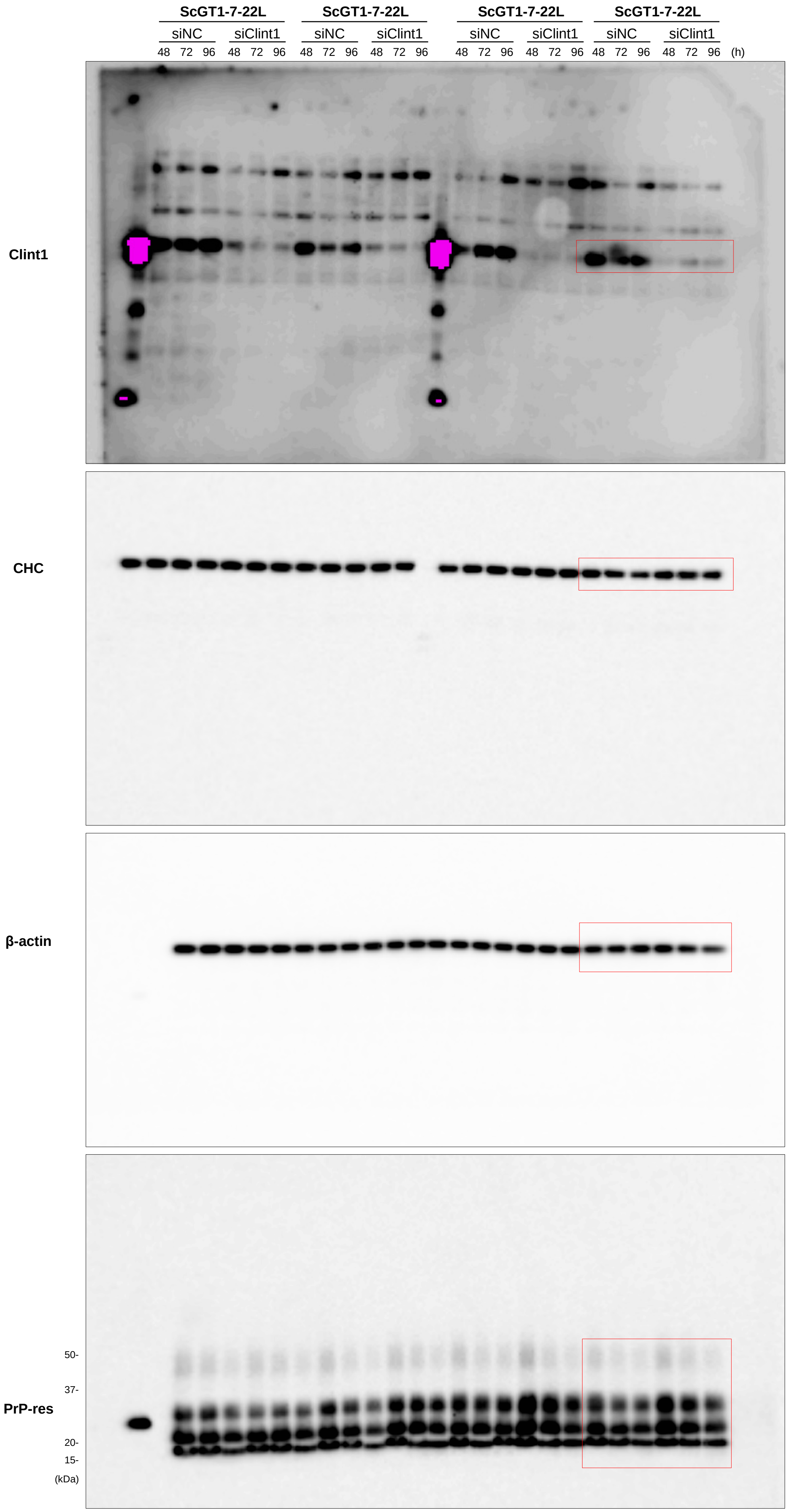

Supplemental Figure S13: Full-length blots of Figure 6. Red-boxed regions are shown in Fig. 6.

Supplementary Figure S14

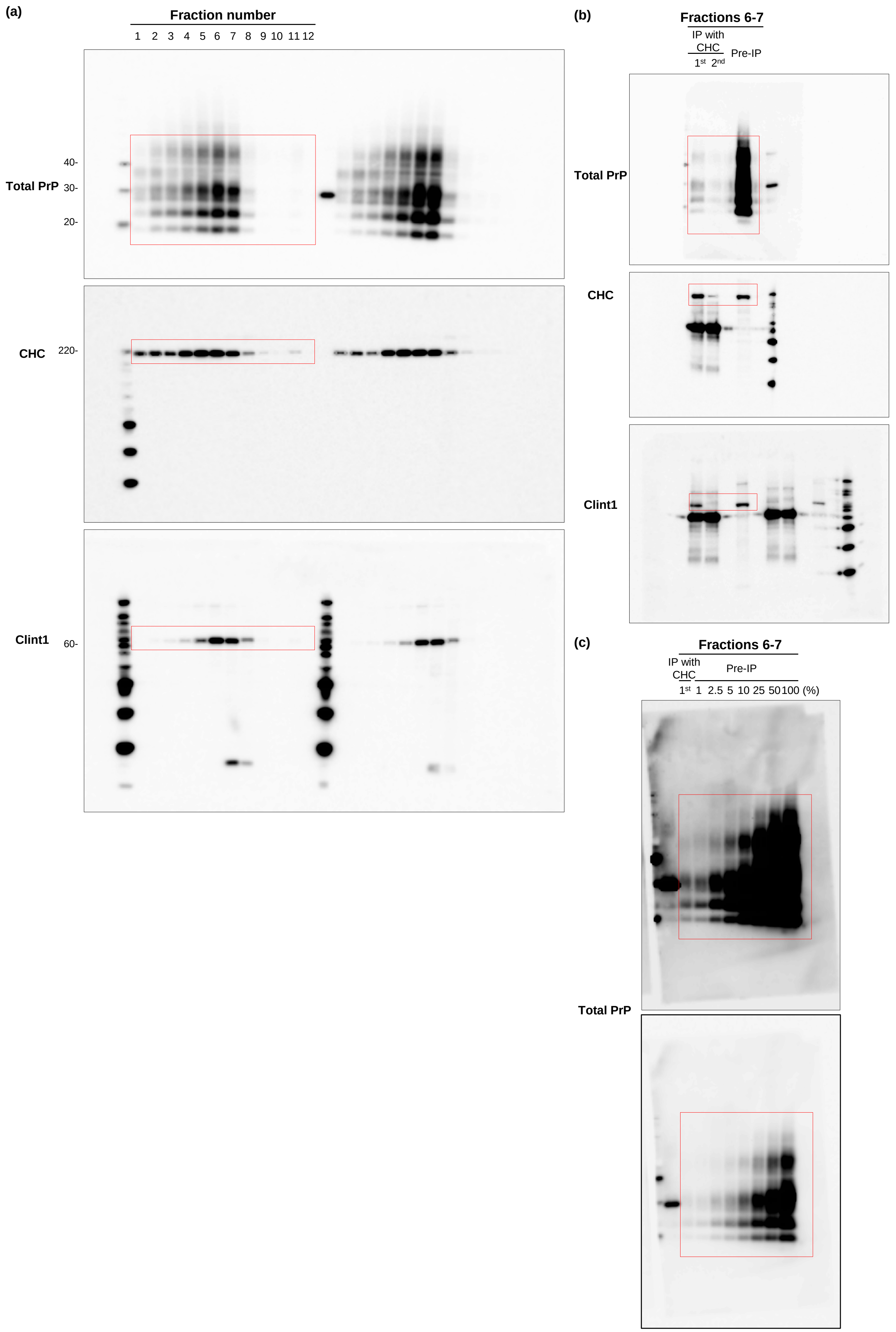

Supplemental Figure S14: Full-length blots of Supplementary Figure S3. Red-boxed regions are shown in Fig. S3.
